# Short-term effects of high-protein, lower-carbohydrate ultra-processed foods on human energy balance

**DOI:** 10.1038/s42255-025-01247-4

**Published:** 2025-03-13

**Authors:** Franziska A. Hägele, Catrin Herpich, Jana Koop, Jonas Grübbel, Rebecca Dörner, Svenja Fedde, Oliver Götze, Yves Boirie, Manfred J. Müller, Kristina Norman, Anja Bosy-Westphal

**Affiliations:** 1 Department of Human Nutrition, Institute of Human Nutrition and Food Science, Kiel University, Kiel, Germany; 2 Department of Nutrition and Gerontology, German Institute of Human Nutrition Potsdam-Rehbrücke, Nuthetal, Germany; 3 Department of Internal Medicine, University Hospital Knappschaftskrankenhaus, Ruhr-University Bochum, Bochum, Germany; 4 Human Nutrition Unit, University of Clermont Auvergne, INRAE, CRNH Auvergne, Auvergne, France; 5 Clinical Nutrition Department, CHU Clermont-Ferrand, Clermont-Ferrand, France; 6 Institute of Nutritional Science, University of Potsdam, Nuthetal, Germany; 7 Department of Geriatrics and Medical Gerontology, Charité-Universitätsmedizin Berlin, Corporate Member of Freie Universität Berlin and Humboldt-Universität zu Berlin, Berlin, Germany; 8 German Center for Cardiovascular Research (DZHK), Partner Site Berlin, Berlin, Germany

**Keywords:** Fat metabolism, Obesity, Pre-diabetes

## Abstract

Protein-enriched ultra-processed foods (UPFs) are generally perceived as a healthy and favourable dietary choice for weight management. However, compared with low-processed foods, the consumption of UPFs has been demonstrated to result in overfeeding and gains in body weight and fat mass. Here we investigate the short-term effects of protein-enriched UPFs on energy intake and energy balance in a single-blind crossover trial involving 21 healthy young adults, who were randomly assigned to 2 UPF diets for 54 hours in a whole-room calorimeter. Participants received either a high-protein (30%) and lower-carbohydrate (29%) diet (HPLC-UPF) or a normal-protein (13%) and normal-carbohydrate (46%) diet (NPNC-UPF). Meals were equally palatable, matched for calories, fat and fibre, and consumed ad libitum. As primary outcomes, compared with NPNC-UPF consumption, the HPLC-UPF diet resulted in a higher energy expenditure (128 ± 98 kcal d^−1^) and lower energy intake (−196 ± 396 kcal d^−1^), leading to a less-positive energy balance (18% versus 32%) with gains in protein and carbohydrate balance only. Postprandial ghrelin levels were lower, whereas glucagon and peptide YY levels were higher with HPLC-UPF compared with NPNC-UPF (secondary outcomes). Despite a reduction in energy intake and increased energy expenditure, the short-term consumption of protein-enriched UPFs did not prevent overeating but did favourably affect energy partitioning. ClinicalTrials.gov registration: NCT05337007.

## Abstract (web summary)

This single-blind crossover trial shows that the short-term consumption of protein-enriched ultra-processed foods does not prevent overeating but favourably affects energy partitioning.

## Main

The dominant share of ultra-processed foods (UPFs) in the global food supply^1^ has shifted the focus of research on the exogenous causes of the obesity pandemic from consumer dietary habits to the composition of industrially processed foods. According to the NOVA classification, UPFs are industrial formulations of low-cost ingredients, often modified by chemical processes with little to no intact unprocessed or minimally processed foods, and the use of cosmetic additives^2^. The underlying mechanisms that cause passive overconsumption of UPFs are proposed to include a high palatability and energy density, as well as the effects of food matrices on eating rate, digestion and metabolism. However, although these characteristics are typical of UPFs, they are not specific to UPFs, as they can also be observed in homemade dishes^3^. Additionally, on average, UPFs tend to exhibit a lower protein content (9.5%, ranging 3–32%) in comparison with processed foods (24.3%, ranging 9–50%)^4^. The lower protein intake with high consumption of UPF was associated with a higher total energy intake, while the absolute protein intake remained relatively constant^4^. This finding is consistent with a basic observation in biology that energy intake varies passively with dietary protein density in different species, as macronutrient regulation of dietary intake minimizes variation in absolute protein intake (‘protein leverage’)^5^.

The food industry is selling an increasing number of more-expensive UPFs labelled as ‘high in protein’^6^ that provide at least 20% of their total energy content from protein^7^. Consumer expectations of these products are high due to advertising health claims on protein and muscle mass or to media claims on satiety and thermogenesis (for a review see ref. ^8^).

Therefore, the present study investigated the effects of 2 days of ad libitum consumption of a high-protein, lower-carbohydrate (HPLC) versus a normal-protein, normal-carbohydrate (NPNC) UPF diet (both 84% UPF) on energy and macronutrient balance, as well as on factors that may explain differences in energy intake (for example eating rate, subjective appetite, gastrointestinal peptide hormones and gastric emptying).

**Extended Data Fig. 1:**
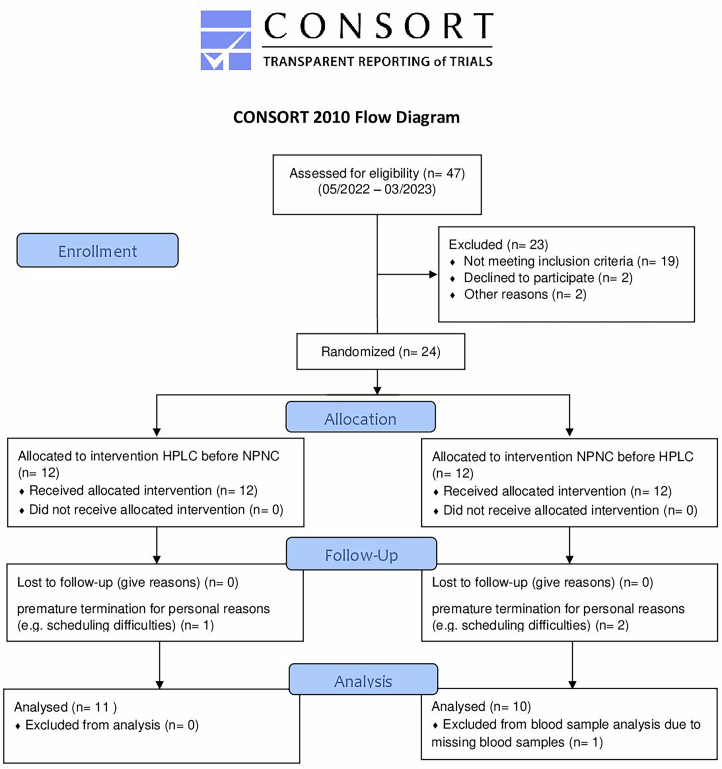
CONSORT 2010 Participant Flow Diagram. HPLC, high-protein, lower-carbohydrate diet; NPNC, normal-protein, normal-carbohydrate diet.

Twenty-four young and healthy participants (13 women and 11 men; Extended Data Table 1) were recruited for this single-blind, crossover, inpatient study in a whole-room indirect calorimeter. Three participants were excluded from the analysis due to premature discontinuation for personal reasons, resulting in a final population of 21 participants (Extended Data Fig. 1).

**Extended Data Fig. 2:**
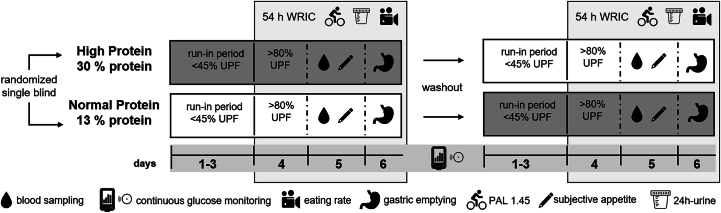
Overview of the study design. Twenty-four healthy young adults participated in a single-blind crossover trial and were randomized to receive either a high-protein, lower-carbohydrate diet (HPLC, 30 % protein, 29 % carbohydrates, CHO) or normal-protein, normal CHO diet (NPNC, 13 % protein, 46 % CHO) for 5.5 days followed by the alternate diet for 5.5 days. Washout between interventions was at least 4 days. Day 1-3 were a run-in period at home with provided foods containing <45 % ultra-processed foods (UPF), whereas days 4-6 comprised a 54-h intervention with >80 % UPF in a whole-room indirect calorimeter (WRIC) at Kiel University. During the inpatient stay, a physical activity level (PAL) of 1.45 was maintained, energy intake and energy expenditure were measured, eating rate was assessed and 24-h urine was collected. On day 5 during breakfast, postprandial blood samples were taken and subjective appetite ratings using visual analogue scales were collected. On day 6 during breakfast, gastric emptying was assessed. Continuous interstitial glucose monitoring was used during the whole study period.

Participants were randomized to receive either the HPLC-UPF diet (30% protein and 29% carbohydrates) or NPNC-UPF (13% protein and 46% carbohydrates) for 5.5 days (days 1–3, run-in with <45% UPF; days 4–6, 54-h intervention with >80% UPF), followed by the alternative diet for 5.5 days (Extended Data Fig. 2). Both diets were of intermediate energy density (1.6–2.4 kcal g^−1^ (ref. ^9^)), consumed ad libitum, designed to be equally palatable and matched for food type, fat and fibre content (Table 1, top, and Methods). Protein intake was 3.3 g kg^−1^ body weight in the HPLC-UPF diet and 1.5 g kg^−1^ body weight in the NPNC-UPF diet (Table 1, bottom).

**Table 1:** Diet composition as provided to the participants (top) and as consumed during the HPLC and NPNC diet periods (bottom)

	Intervention days		Run-in period	
	HPLC	NPNC	HPLC	NPNC
**Diet as provided**				
Protein (%E)	29.3 ± 0.7	13.4 ± 0.7	30.0 ± 0.2	13.3 ± 0.1
Carbohydrates (%E)	29.9 ± 3.4	46.4 ± 3.0	28.5 ± 0.8	45.9 ± 0.7
Fat (%E)	38.6 ± 2.3	37.3 ± 2.0	37.6 ± 0.3	37.9 ± 0.1
SFA (%E)	41.2 ± 2.5	44.6 ± 2.1		
MUFA (%E)	38.2 ± 2.4	40.5 ± 2.5		
PUFA (%E)	19.5 ± 1.2	13.5 ± 0.2		
Fibre (%E)	2.3 ± 0.2	2.8 ± 0.4	3.9 ± 0.8	3.0 ± 0.6
Ultra-processed (%E)^a^	84.3 ± 7.2	84.5 ± 5.6	41.4 ± 13.3	39.0 ± 16.8
**Diet as consumed**				
Energy (kcal d^−1^)	3,225 ± 922	3,421 ± 922*	2,465 ± 672	2,832 ± 688***
Protein (%E)	30.4 ± 0.3	13.1 ± 1.0***	27.0 ± 1.1	11.5 ± 0.5***
Carbohydrates (%E)	29.3 ± 1.1	46.1 ± 3.0***	29.2 ± 1.0	43.3 ± 2.0***
Fat (%E)	37.7 ± 0.6	38.3 ± 1.6	39.2 ± 1.5	42.1 ± 2.1***
Fibre (%E)	2.6 ± 0.3	2.6 ± 0.5	4.7 ± 0.2	3.1 ± 0.2***
Fibre (g d^−1^)	42 ± 12	44 ± 13	57 ± 17	42 ± 11***
Sugar (g d^−1^)	79.0 ± 26.3	148.6 ± 42.9***	90.5 ± 21.6	103.2 ± 24.4***
Salt (g d^−1^)	11.7 ± 3.3	11.0 ± 3.1	7.3 ± 1.9	7.1 ± 1.9
Protein (g kg^−1^ BW)	3.3 ± 0.7	1.5 ± 0.3***	2.2 ± 0.5	1.1 ± 0.2***
Energy density (heated) (kcal g^−1^)	1.94 ± 0.09	2.03 ± 0.08***		

^a^The calculated energy percentage refers to the fraction of diet calories contributed from group 4 of the NOVA classification system: (1) unprocessed or minimally processed, (2) processed culinary ingredients, (3) processed foods and (4) UPFs.

Values are mean ± s.d. and *P* values (‘diet as consumed’ data) are from a paired two-sided *t*-test or Wilcoxon test comparing high-protein and normal-protein diet. *P < 0.05, ***P < 0.001. %E, energy per cent; BW, body weight; SFA, saturated fatty acid; MUFA, mono-unsaturated fatty acid; PUFA, poly-unsaturated fatty acid.

**Fig. 1:**
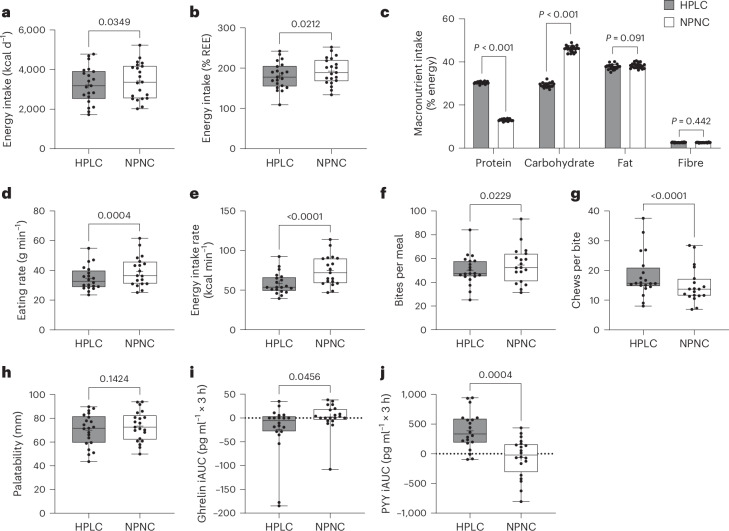
Ad libitum food intake, eating rate and appetite-related hormones. **a**,**b**, Ad libitum daily energy intake (**a**, absolute in kcal d^−1^; **b**, relative in % of resting energy expenditure) was lower with the HPLC-UPF diet (30% protein and 29% carbohydrates) compared with the NPNC-UPF diet (13% protein and 46% carbohydrates). REE, resting energy expenditure. **c**, Intake of protein was higher and intake of carbohydrates was lower with the HPLC-UPF compared with the NPNC-UPF, whereas intake of fat and fibre did not differ. **d**,**e**, Eating rate (**d**) and energy intake rate (**e**) were lower with HPLC-UPF compared with NPNC-UPF (both *n* = 18). **f**,**g**, Bites per meal (**f**) were lower and chews per bite (**g**) were higher with HPLC-UPF compared with NPNC-UPF (both *n* = 18). **h**, Both diets were rated equally palatable on visual analogue scales (*n* = 20). **i**,**j**, During breakfast on day 5, ghrelin secretion (**i**) was suppressed, whereas PYY secretion (**j**) was increased with HPLC-UPF compared with NPNC-UPF (both *n* = 20). All box plots show the interquartile range with the 25% (lower hinge), 50% (centre line) and 75% (upper hinge) quantiles. Whiskers extend to the minimum and maximum values. For parametric data, the mean is displayed as +. Data in the bar graphs are presented as mean ± s.d. (**c**). *n* = 21 unless stated otherwise. *P* values were from paired two-sided *t*-tests (**a**–**f** and **h**–**j**) or Wilcoxon tests (**g**). iAUC, incremental area under the curve. Source data

Ad libitum consumption of HPLC-UPF resulted in a 196 ± 396 kcal d^−1^ lower energy intake compared with NPNC-UPF (Fig. 1a,b and Table 1, bottom). Meal duration was minimally longer with HPLC-UPF compared with NPNC-UPF (*P* < 0.05). With HPLC-UPF, eating rate (*P* < 0.001) and energy intake rate (*P* < 0.001) were slower, fewer bites per meal were taken (*P* < 0.05) and more chews per bite were performed (*P* < 0.0001) compared with NPNC-UPF (Fig. 1d–g). The lower energy intake with HPLC-UPF may be explained by a slower eating rate because previous studies have shown that a higher eating rate contributes to increased energy intake^10,11^ and is influenced by food texture, with softer foods being consumed more quickly^10^. Textural profile analysis has shown that food protein content is associated with springiness and chewiness, which lead to a slower eating rate^12^. Although not measured for the study diets, these data suggest that the textural properties of foods rich in protein may have contributed to reduced consumption rates in the HPLC-UPF diet.

Accordingly, the 3-h postprandial ghrelin levels were lower (*P* < 0.05; Fig. 1i) and peptide YY (PYY) levels were higher (*P* < 0.001; Fig. 1j) in HPLC-UPF compared with the NPNC-UPF condition. This effect may be due to prolonged oro-sensory exposure induced by a slower eating rate, which has been shown to stimulate the release of appetite-related hormones^13^. Similar to our results, some studies^14,15^ but not all^16,17^ have shown lower postprandial ghrelin secretion with a high-protein diet compared with a high-carbohydrate diet. The lower carbohydrate content^18^ together with the amino acid composition of a mixed meal^19^ contribute to the postprandial increase in glucagon secretion, which acts in the liver to reduce food intake via vagus nerve signalling to the brain^20^. In addition, increased secretion of the anorexigenic PYY by a high-protein diet^15,21^ has been found to play a critical role in protein-mediated satiety^22^.

**Extended Data Fig. 3:**
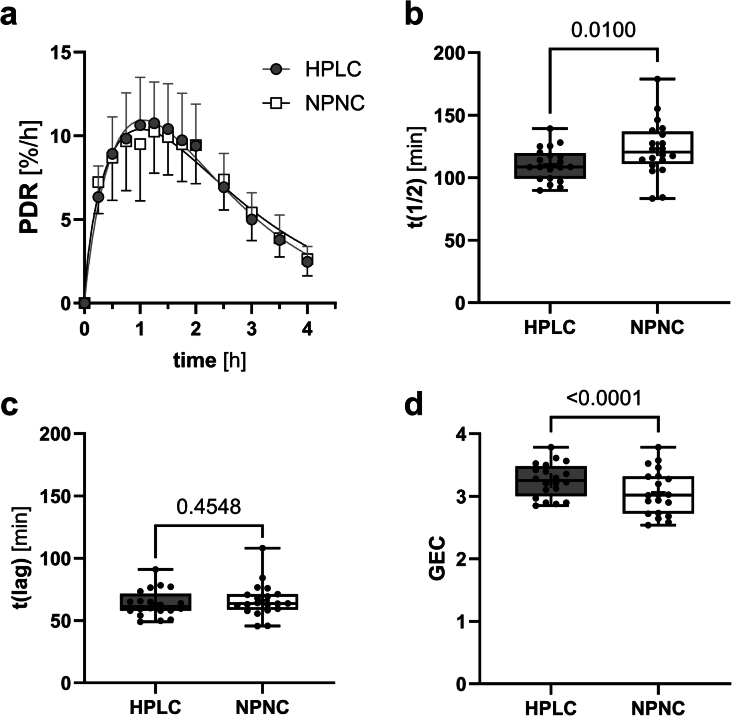
Gastric emptying following an isocaloric test meal using a ^13^C-breath test. Gastric emptying was assessed during 4 h following an isocaloric breakfast (porridge, 25 % of individual REE) on day 6 either with a high-protein content (HP-UPF, 30 % protein, 47 % carbohydrates, CHO, 18 % fat, 4 % fibre) or a normal-protein content (NP-UPF,13 % protein, 64 % CHO, 18 % fat, 4 % fibre) of ultra-processed foods (UPF). Percentage ^13^C-dose recovery per hour (**a**) was similar for HP-UPF and NP-UPF. Gastric half-emptying time (t_1/2_, **b**) was lower with HP-UPF compared to NP-UPF, whereas gastric lag time (t_lag_, **c**) was similar for both interventions and the gastric emptying coefficient (GEC, **d**) was higher for HP-UPF compared to NP-UPF. All box plots show the interquartile range with the 25 % (lower hinge), 50 % (centre line), and 75 % (upper hinge) quantiles. Whiskers extend to the minimum and maximum values. For parametric data, the mean is displayed as +. Data in diagram are presented as mean ± SD (a). N = 20, p-values from paired two-sided t-tests. HPLC, high-protein, lower-carbohydrate diet; NPNC, normal-protein, normal-carbohydrate diet.

Notably, the lower food intake with HPLC-UPF occurred despite faster gastric emptying during a high-protein versus normal-protein test meal (Extended Data Fig. 3a–d). The percentage of ^13^C-dose recovery per hour and the gastric emptying delay time were similar for both meals whereas the gastric half-emptying time was shorter (*P* < 0.05) and the gastric emptying coefficient was higher with the high-protein meal (*P* < 0.0001). These findings are in contrast to the prevailing view that a high-protein content promotes satiety in part by slowing gastric emptying via increased secretion of GLP-1, PYY and glucagon and decreased secretion of ghrelin^23^. Thus, the observed slower gastric emptying with the normal-protein test meal is likely due to a higher osmolarity with higher carbohydrate and sugar content^24,25^.

To achieve a similar energy density of the diets, macronutrient intake differed between the HPLC-UPF and NPNC-UPF interventions for protein (30% versus 13%, *P* < 0.001; Fig. 1c and Table 1, bottom) and carbohydrate (29% versus 46%, *P* < 0.001; Fig. 1c and Table 1, bottom). Still, the energy density was slightly higher in NPNC-UPF compared with HPLC-UPF diets when comparing the heated foods (*P* < 0.001; Table 1, bottom). As fat and fibre content did not differ between diets (Fig. 1c and Table 1, bottom), this discrepancy was likely due to greater water vapour loss after heating. Nevertheless, the subtle discrepancies in energy density are unlikely to account for the differences in energy intake, as other researchers have demonstrated that the correlation between meal energy density and caloric intake is nonlinear, exhibiting an increase with rising energy density up to approximately 1.5 kcal g^−1^, followed by a slight decline^26^.

**Fig. 2:**
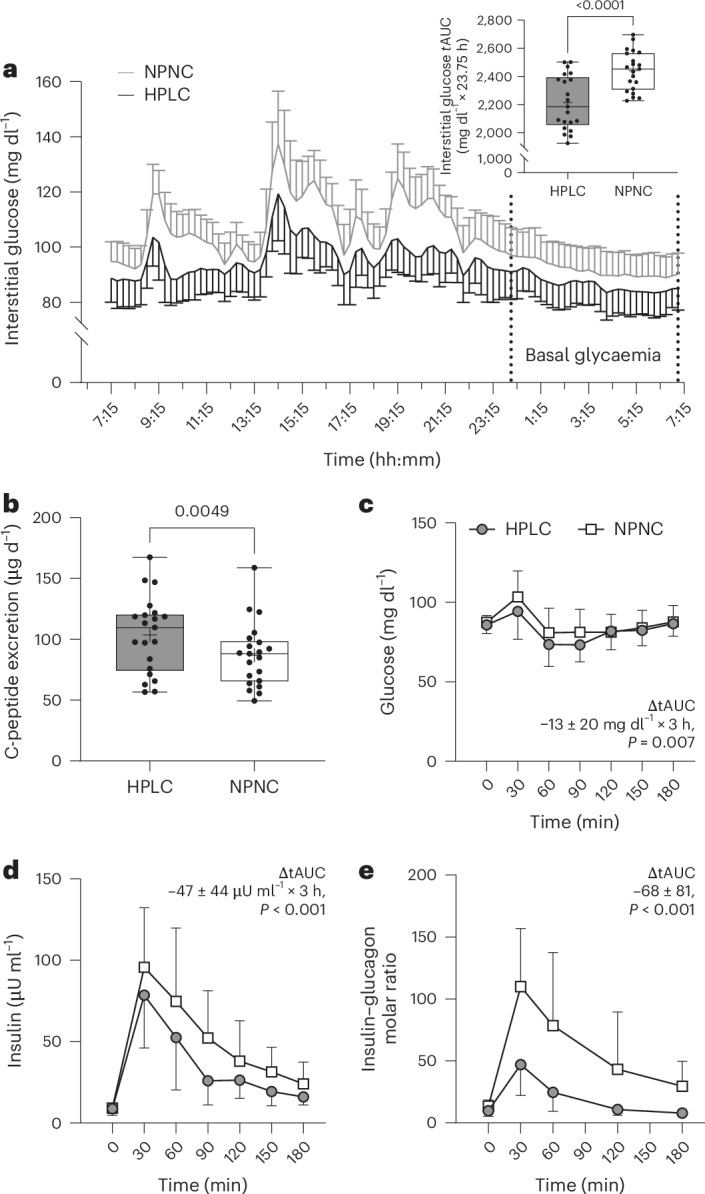
Diurnal and postprandial glycaemia. **a**,**b**, Diurnal glycaemia (continuous interstitial glucose monitoring) throughout the inpatient intervention (**a**) was higher with the NPNC-UPF diet (13% protein and 46% carbohydrates) compared with the HPLC-UPF diet (30% protein and 29% carbohydrates), while 24-h-insulin secretion (measured as C-peptide excretion) was higher with HPLC-UPF compared with NPNC-UPF (**b**). **c**,**d**, Following breakfast on day 5, postprandial levels of glucose (**c**) were similar between interventions, whereas insulin levels (**d**) were lower with HPLC-UPF compared with NPNC-UPF (both *n* = 20). **e**, The molar insulin to glucagon ratio was higher with NPNC-UPF compared with HPLC-UPF (*n* = 20). All box plots show the interquartile range with the 25% (lower hinge), 50% (centre line) and 75% (upper hinge) quantiles. Whiskers extend to the minimum and maximum values. For parametric data, the mean is displayed as +. Data in diagrams are presented as mean ± s.d. (**a** and **c**–**e**). *n* = 21 unless stated otherwise. *P* values were from paired two-sided *t*-tests (**a**–**c**) or Wilcoxon tests (**d** and **e**). tAUC, total area under the curve. Source data

**Fig. 3:**
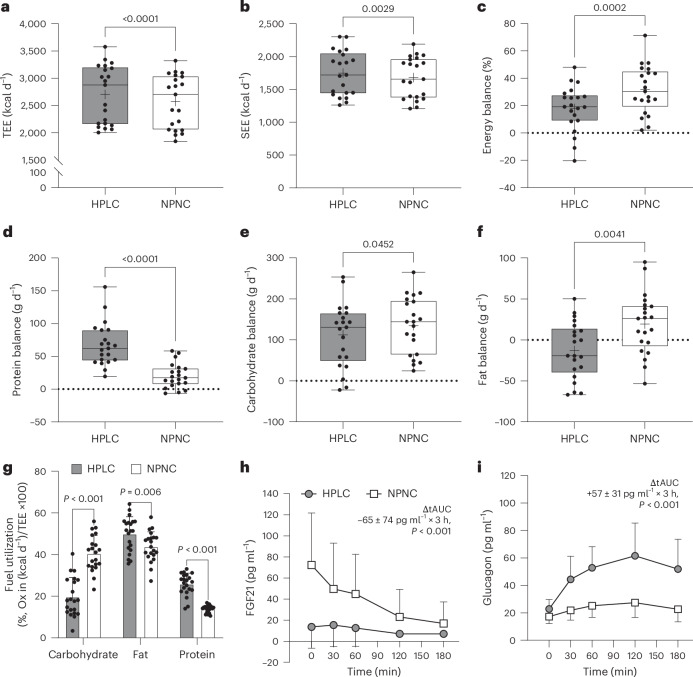
Energy expenditure, energy and macronutrient balances and hormones. **a**,**b**, Total energy expenditure (TEE) (**a**) and sleeping energy expenditure (SEE) (**b**) were higher with the HPLC-UPF diet (30% protein and 29% carbohydrates) compared with the NPNC-UPF diet (13% protein and 46% carbohydrate). **c**, Energy balance was lower with HPLC-UPF compared with NPNC-UPF, although positive with both interventions (both *P* < 0.001). **d**–**f**, Protein balance (**d**) was more positive with HPLC-UPF compared with NPNC-UPF and carbohydrate balance (**e**) was similar between interventions, whereas fat balance (**f**) was positive with NPNC-UPF compared with HPLC-UPF. **g**, Fuel utilization (macronutrient oxidation in % of 24-h energy expenditure) was lower for carbohydrate and higher for fat and protein with HPLC-UPF compared with NPNC-UPF. Ox, oxidation. **h**, Fibroblast growth factor 21-secretion (FGF21) was reduced with HPLC-UPF and high with NPNC-UPF and decreased postprandially after breakfast on day 5 with NPNC-UPF (*n* = 20). **i**, Glucagon secretion increased postprandially more pronounced with HPLC-UPF compared with NPNC-UPF (*n* = 20). All box plots show the interquartile range with the 25% (lower hinge), 50% (centre line) and 75% (upper hinge) quantiles. Whiskers extend to the minimum and maximum values. For parametric data, the mean is displayed as +. Data are presented as mean ± s.d. (**h** and **i**). *n* = 21 unless stated otherwise. *P* values are from paired two-sided *t*-tests (**a**–**c**, **e**–**g** and **i**) or Wilcoxon tests (**d** and **h**). Source data

The effects of HPLC-UPF on energy intake could also be explained by the lower carbohydrate content of the diet, as a high glycaemic load could lead to a positive energy balance through higher insulin and lower glucagon secretion^27^. However, this is an unlikely explanation because both 24-h-insulin (*P* < 0.01; Fig. 2b) and postprandial glucagon secretion (*P* < 0.001; Fig. 3i) were higher in the HPLC-UPF diet.

Although glucagon stimulates gluconeogenesis^28^, basal and diurnal glycaemia were lower with the HPLC-UPF diet (both *P* < 0.01; Fig. 2a), presumably because the amino acids stimulated 24-h insulin secretion, as evidenced by higher C-peptide excretion. Postprandial glucose (*P* < 0.01; Fig. 2c) and insulin levels (*P* < 0.001; Fig. 2d) were both lower in HPLC-UPF compared with NPNC-UPF. The postprandial insulin to glucagon molar ratio was lower in HPLC-UPF compared with NPNC-UPF (*P* < 0.001; Fig. 2e) reflecting the combined effects of both hormones on hepatic metabolism with a shift to glucagon in response to HPLC-UPF^29^. Taken together, the improved glycaemic control during HPLC-UPF, despite a high caloric intake and high glucagon levels, suggests that increased insulin secretion in this situation does not reflect insulin resistance.

In a representative study from the United States, the top quintile of UPF consumption was 85% (ref. ^30^); therefore, our study reflects these values. During the 3-day run-in phase we used a <45% UPF ad libitum diet, which is roughly similar to the average UPF intake in Germany at 46% (ref. ^31^). This amount of UPF has been shown to be well within the range that could drive an obesity epidemic^32^. Compared with the HPLC-UPF effect, the HPLC diet in the run-in phase resulted in a greater reduction in energy intake (−196 ± 396 kcal d^−1^ versus −367 ± 264 kcal d^−1^; *P* < 0.05; Table 1, bottom). This suggests that the protein leverage is more pronounced at moderate UPF consumption, but may be outweighed by factors that facilitate UPF overconsumption (for example energy density, hyperpalatability and soft texture) at a high UPF diet. The large difference in energy intake between medium and high UPF consumption within the high-protein intervention (high-protein run-in versus HPLC-UPF −760 ± 464 kcal d^−1^) may not be due to overconsumption of high-protein UPFs, but to an even lower consumption of high-protein foods in the run-in phase due to a higher fibre intake (*P* < 0.05; Table 1, bottom). It can, thus, be assumed that the greater difference in energy intake with the high-protein intervention is not only a protein leverage effect but also due to a higher fibre content (and texture).

The 24-h and sleep energy expenditure were both higher in HPLC-UPF compared with NPNC-UPF (+128 ± 98 kcal d^−1^; *P* < 0.001; Fig. 3a; +67 ± 90 kcal d^−1^; *P* < 0.01; Fig. 3b). Physical activity level was similar between interventions with 1.46 ± 0.08 for HPLC-UPF and 1.45 ± 0.06 for NPNC-UPF (*P* > 0.05). The higher 24-h energy expenditure with HPLC-UPF confirms the results of previous studies with mildly hypocaloric high-protein diets (+82 kcal d^−1^ (ref. ^33^); +120 kcal d^−1^ (ref. ^34^)). This effect can be partially explained by a high diet-induced thermogenesis with protein intake due to the metabolic cost of amino acid absorption and metabolism and a high-protein turnover rate^35^. Concomitantly, the 3-h postprandial glucagon levels were higher with HPLC-UPF compared with NPNC-UPF (*P* < 0.001; Fig. 3i). The higher glucagon secretion with HPLC-UPF may have contributed to an increase in energy expenditure via glucagon-induced gluconeogenesis and ureagenesis, reflecting an increased hepatic utilization of amino acids^28^. However, acute effects of glucagon infusion in humans suggest that the stimulation of energy expenditure is high in the fasting state and diminishes in the postprandial state and with insulin infusion (for a review, see ref. ^36^). In our study, the increase in 24-h energy expenditure occurred despite voluntary overfeeding and higher 24-h insulin secretion. Therefore, discrepant results may be due to the high load of amino acids and/or an interaction with other incretins, which may potentiate the effects of glucagon on energy expenditure, and are not observed under the experimental conditions of isolated glucagon infusion. The stimulation of amino acid utilization in the liver by glucagon^28^ is reflected by the positive relationship between nitrogen excretion (resembling urea production) and glucagon levels in HPLC-UPF (*r* = 0.62, *P* < 0.01). In addition, futile cycling in liver metabolism resulting from catabolic effects of glucagon and the anabolic action of insulin^37^ as well as cortisol and thyroid hormones may all have contributed to the thermic effect of a HPLC-UPF diet (for a review see ref. ^36^).

Finally, our finding of an increase in sleep energy expenditure with high-protein UPF compared with NPNC-UPF confirms findings from previous studies with high-protein diets^33,34^ and argues against a purely postprandial phenomenon. A lower ratio of energy balance to nitrogen balance, reflecting a higher protein turnover rate, may promote higher basal and sleeping energy expenditure with high-protein diets^38^.

Due to the high-protein content of the diet during the previous four days, fasting FGF21 levels were lower with HPLC-UPF compared with NPNC-UPF (*P* < 0.001; Fig. 3h). Higher fasting FGF21 levels with NPNC-UPF allowed a decrease in the postprandial phase (as shown previously^39^), whereas with HPLC-UPF postprandial FGF21 secretion was already maximally suppressed.

Energy balance was positive for both diets with >80% UPF, consistent with the findings of Hall et al. who found that a diet containing 83% UPF within similar food groups of a contemporary western diet for 14 days resulted in an increase in body weight and fat mass^11^. However, the energy balance was less positive with HPLC-UPF compared with NPNC-UPF (+18% versus +32% *P* < 0.001; Fig. 3c). Although the lower energy intake with HPLC-UPF corresponds to the phenomenon of ‘protein leverage’^4^, ad libitum intake of HPLC-UPF could not prevent overfeeding even at very high protein intakes (>3 g kg^−1^ body weight). This suggests that the higher eating rate of UPF and a relatively high energy density may ameliorate the protective effects of a high-protein intake on weight gain.

Protein oxidation was higher (+90 ± 42 g d^−1^; *P* < 0.001) and protein balance was more positive with HPLC-UPF compared with NPNC-UPF (*P* < 0.001; Fig. 3d). In contrast, carbohydrate oxidation was lower (−131 ± 74 g d^−1^; *P* < 0.001) and fat oxidation was higher (+24 ± 32 g d^−1^; *P* < 0.01) with HPLC-UPF, resulting in a less-positive carbohydrate balance (*P* < 0.05; Fig. 3e). By contrast, fat balance was positive with NPNC-UPF only (one sample *t*-test, +19%; *P* < 0.05; Fig. 3f) and equal with HPLC-UPF (one sample *t*-test, −6%; *P* = 0.297). Overconsumption of HPLC-UPF resulted in higher fat and protein and lower carbohydrate utilization (Fig. 3g). As postprandial glucagon secretion was higher with HPLC-UPF, the ability of glucagon to promote lipid catabolism over storage^40^ may have contributed to this effect. Previous studies of mildly hypocaloric high-protein diets (30% protein, 40% carbohydrate and 30% fat^34^ and 40% protein, 35% carbohydrate and 25% fat^33^) also found higher protein and fat, as well as a lower-carbohydrate oxidation rate resulting in negative fat balances and increased protein anabolism compared with normal-protein diets (10% protein, 60% carbohydrate, 30% fat^34^ and 15% protein, 55% carbohydrate, 30% fat^33^). In support of the findings from short-term protocols, 8 weeks of overfeeding (+ 40%) with varying protein intake (5–25%) predicted the increase in lean body mass but not the change in fat storage^41^.

Our study has some limitations. HPLC-UPF foods had a lower sugar content (−69.6 ± 23.3 g d^−1^; *P* < 0.001; Table 1, bottom). Although not measured for the study diets, high-protein UPFs on the German market are typically higher in artificial sweeteners and flavourings^42^. This may partly compensate for the lower palatability of these foods. Consistent with this, both diets were rated as equally palatable (*P* > 0.05; Fig. 1h). In comparison to artificial sweeteners, sugars have been demonstrated to induce satiety and reduce food intake in the short term^43^. When compared with an equivalent quantity of starch, sugars do not elicit a more pronounced elevation in blood glucose and insulin levels because fructose and sucrose exhibit a markedly lower glycaemic index, with values that are up to 50% lower than those of the most prevalent starchy foods^44^. However, we cannot exclude the possibility that differences in sugar content between the diets contribute to the differences in glycaemia and energy partitioning.

**Extended Data Fig. 4:**
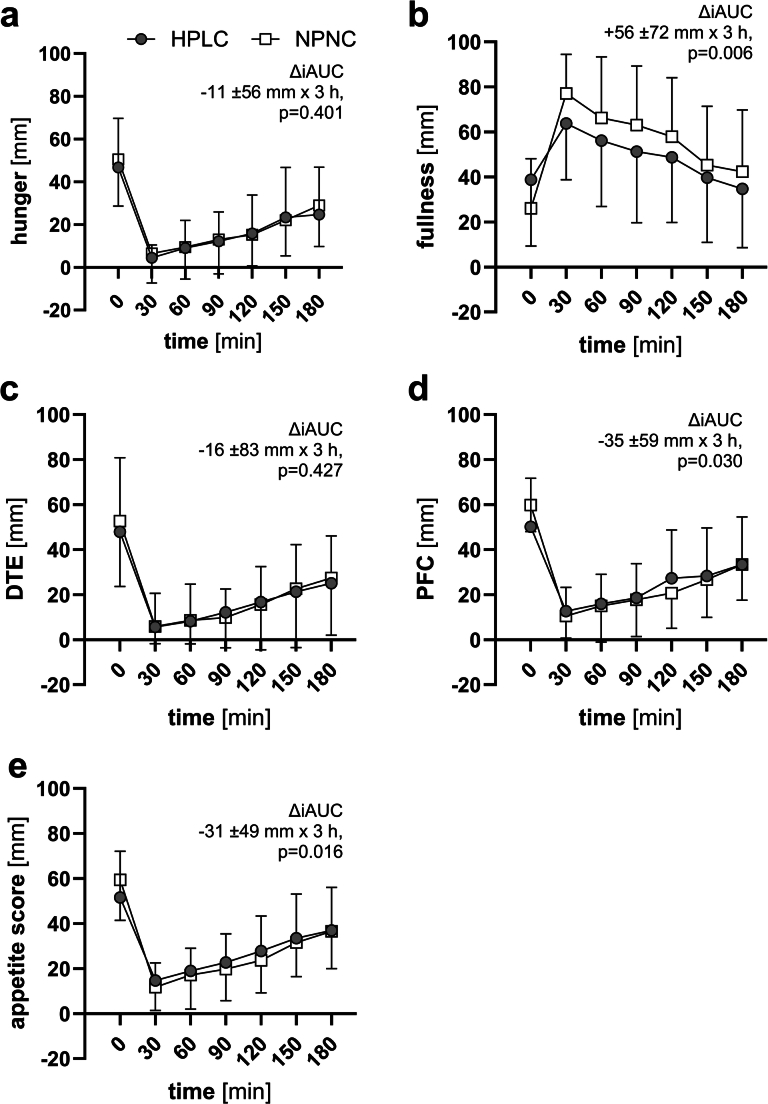
Subjective appetite perceptions. Subjective appetite perceptions were assessed during 3 h following breakfast on day 5 using visual analogue scales. Perceived hunger (**a**) and desire to eat (DTE, **c**) after ad libitum energy intake were similar with the high-protein, lower-carbohydrate-ultra-processed food diet (HPLC-UPF, 30 % protein, 29 % carbohydrates, CHO) compared to the normal-protein, normal-carbohydrate diet (NPNC-UPF, 13 % protein, 46 % CHO). Subjective fullness (**b**, n = 18) was lower and prospective food consumption (PFC, **d**) was higher with HPLC compared to NPNC. Correspondingly the subjective appetite score (**e**, mean from ratings of hunger, fullness, desire to eat and prospective food consumption) was higher with HPLC-UPF compared to NPNC-UPF. Data are presented as mean ± SD. N = 19 unless stated otherwise, p-values from paired two-sided t-tests. iAUC, incremental area under the curve.

We found a lower 3-h subjective appetite score after an ad libitum meal with NPNC-UPF compared with HPLC-UPF (*P* < 0.05), which seems contradictory. However, the appetite score is the mean of ratings of hunger, fullness, desire to eat and prospective food consumption (Extended Data Fig. 4a–e). The lower appetite score with NPNC-UPF was only due to higher perceived fullness (*P* < 0.01) and lower prospective food consumption (*P* < 0.05), which seem to be reasonable results, given that a greater amount of food was consumed with NPNC-UPF (higher energy intake for the same energy density). As the appetite score does not cover the full 5-h period until the next meal, it does not argue against increased energy intake at lunch and dinner with NPNC-UPF. The finding of a lower appetite score with NPNC-UPF also contradicts the PYY and ghrelin results, which indicate a more-satiating effect of HPLC-UPF. While many studies found that high protein intake could suppress subjective appetite sensations more, other studies also did not find this effect (see ref. ^15^ for a discussion). In our study, the interpretation of the changes in hormones and appetite score may be limited by the non-isocaloric test conditions.

As a further limitation of our study, we investigated only short-term effects of dietary protein content on energy balance (a 3-day run-in period followed by a 54-h intervention with UPF). A long-term HPLC-UPF diet that does not prevent overfeeding may increase the risk of insulin and glucagon resistance in people with overweight. In the PROOF study, 8 weeks of ~40% overfeeding with high protein (25% protein) compared with normal protein (15% protein) resulted in a higher increase in intrahepatic lipid content^45^. In addition, prospective population studies have found a correlation between a high protein intake and an increased risk for prediabetes and type 2 diabetes.

In conclusion, despite a reduction in energy intake and an increase in energy expenditure, short-term consumption of HPLC-UPF was ineffective in preventing overeating. Further investigation is required to elucidate the long-term effects of HPLC-UPF on cardiometabolic risk in vulnerable populations with overweight or prediabetes.

## Methods

### Study population

Twenty-four healthy adults (13 women and 11 men) between the ages of 18 and 35 years with a body mass index (BMI) between 19 and 29 kg m^−2^ and a low-to-normal level of habitual physical activity were recruited and data were collected from May 2022 to April 2023 at the Kiel University, Germany using a noticeboard and social media postings, information on the website of Kiel University and by contacting former study participants who consented to this. The objective was to achieve an equal distribution of sexes based on self-reports. Women were included with a regular menstrual cycle only and during their follicular phase to avoid influences of the female cycle on energy expenditure^46^ and appetite. Exclusion criteria were chronic diseases (including renal dysfunction), regular use of medication, alternative eating habits (such as being vegan or vegetarian), food allergies or intolerances, claustrophobia, smoking, high habitual physical activity (≥1 h d^−1^), current weight loss diet or weight loss of >5 kg in the last 3 months, pregnancy or lactation. Before their enrolment in the study, participants were presented with the proposed meal plans. Only those who expressed a willingness to adhere to the specified dietary regimen were included in the study. The participants were not informed of the true aim of the study (single-blind), but were told that the study was investigating the effect of differences in protein content on energy expenditure. The study protocol was approved by the ethics committee of the Medical Faculty at Kiel University (D456/22) in accordance with the Declaration of Helsinki. This trial was registered at ClinicalTrials.gov as NCT05337007. All participants provided written informed consent before participation.

Participants were invited to attend an in-person screening, which was conducted within 2 weeks before the interventions after an overnight fast. Height was measured with a stadiometer and body weight was assessed on a scale (seca 274; seca). Resting energy expenditure (REE) was measured for 20 min via indirect calorimetry using a canopy hood (Q-NRG, COSMED) to determine the amount of provided food for the run-in periods. To confirm participation in the study, blood samples were collected for the measurement of ferritin and creatinine concentrations and urine samples were obtained for the examination of albumin. The German version of the Three*-*Factor Eating Questionnaire was used to exclude restraint eaters^47^. Ethnicity was self-determined by participants. Three female participants were excluded from the study due to premature termination for personal reasons (such as scheduling difficulties). The final population consisted of 21 participants (10 females and 11 males). A CONSORT (Consolidating Standards for Reporting Trials) flow diagram of participants is available in Extended Data Fig. 1.

### Study protocol

The randomized single-blind crossover trial comprised a highly controlled nutritional intervention with two 54-h stays in a whole-room indirect calorimeter (WRIC; preceded by 3 days of run-in each) at the Institute of Human Nutrition, Kiel University. Two interventions at a physical activity level (PAL) of 1.45 were conducted: (1) NPNC diet and (2) HPLC diet. An outline of the study protocol is given in Extended Data Fig. 2. The sequence of NPNC and HPLC intervention was randomized through block randomization, with computer-generated random numbers and a block size of 12. The randomization was stratified by sex and the allocation to WRIC 1 or 2. The participants were not made aware of the specific sequence in which they would receive the interventions (single-blind design).

During the interventions, a PAL of 1.45 was obtained by cycling 3 × 20 min per day (in total 60 min d^−1^) on a bicycle ergometer (opticare basic and ergoselect 4, ergoline) while participants were inactive (mainly sitting or lying but awake) for the remaining time of the day. Women were requested to cycle at 50 W and men at 75 W with a constant cadence (55–65 rpm).

To ensure a consistent baseline and to facilitate the adaptation of macronutrient oxidation to macronutrient intake, a controlled diet with a fixed macronutrient composition was implemented for a 3-day run-in period before each intervention period^48^. After an overnight fast and assessment of body composition, participants entered the WRIC at approximately 7:30 on the initial intervention day per period. The following 54-h intervention period comprised 2.5 days and 2 nights. The daily routine was strictly controlled, with a wake-up at 7:15, breakfast at 8:30, first activity bout at 11:45, lunch at 13:30, second activity bout at 16:45, dinner at 18:30, snack opportunity between 20:00 and 21:15, third activity bout at 21:45 and bedtime at 22:45 for days 4 and 5 within each intervention. Following the wake-up at 7:15, the sixth day of the study comprised a test meal as breakfast at 8:30, followed by the assessment of gastric emptying via ^13^C-breath test over 4 h. Washout between the 54-h interventions in the WRIC was at least 4 days.

### Diet composition

The diet during run-in periods consisted predominantly of foods other than UPFs (<45% energy from UPFs) resembling the average intake of UPFs in Germany. In contrast, the diet consumed during the intervention days was primarily composed of UPFs (>80% energy from UPFs; Table 1, top). All meals were provided in excess during the run-in periods (energy provided = REE × PAL 1.8) and on ad libitum energy intake (EI) days in the WRIC (energy provided = REE × PAL 1.4 × 2). To prevent restricted eating due to a limitation in the availability of food, twice the expected energy expenditure has been provided during the WRIC phase. During the run-in phase, the energy provided was calculated as 1.8 × REE which was sufficient to meet the energy requirement, given the prescribed restrictions on physical activity (no exercise).

On intervention days in the WRIC, the diet consisted of the same food items comprising regular UPF items for NPNC and the same UPF items promoted as high-protein versions for HPLC (Supplementary Figs. 1 and 2), blinding the participants to their individual intervention sequence. Comparable palatability of the diets was facilitated through previous tasting of the products by the study team.

The mean macronutrient composition per diet of three daily meals (with an optional snack on days 4 and 5) for the intervention days and run-in period is shown in Table 1, top. Diets were matched for fat content rather than carbohydrate (CHO) content to avoid substantial differences in energy density, which are well known to impact ad libitum EI. In addition, our pre-study on the characteristics of high-protein UPFs revealed that on the German market these products are typically characterized by the replacement of CHO with protein^42^. The macronutrient composition (including fatty-acid patterns) of the provided diet on the intervention days in the WRIC was analysed in pooled food samples per day by an accredited and certified food analysis laboratory (AgroLab LUFA). For the run-in period, the macronutrient composition of the provided diet was calculated using PRODI expert software (v.6.12, Wissenschaftliche Verlagsgesellschaft). Fibre was not matched in this period, as the excessive use of animal protein and plant-based meat alternatives should be avoided, both of which are relatively low in fibre. Thus, the fibre content was higher during HPLC in the run-in phase due to the inclusion of soy, lentils and chickpeas.

On days 4 and 5, participants were offered an optional snack that was matched for macronutrient composition following the evening meal. Participants were instructed to consume all meals within a 30-min time frame and to ingest an equivalent quantity of each food item to maintain a consistent macronutrient composition until they felt comfortably full. The leftovers were weighed and the energy and macronutrient intake were calculated. During the consumption of meals, the use of media was prohibited to prevent the occurrence of distracted eating.

On the sixth day of the intervention period, the test meal was isocaloric, corresponding to 25% of the individual REE, and had to be completed within a time frame of 5–10 min without any remaining food. The protein levels consumed during the WRIC period were replicated in the test meal with the fat and fibre levels being matched between test meals. The macronutrient composition of the test meal was for normal protein: 13.4% protein, 64.4% CHO, 17.8% fat and 4.4% fibre; and for high protein: 30.3% protein, 47.2% CHO, 17.8% fat and 4.4% fibre.

To ensure the consistency and standardization of the meals prepared for the duration of the study, commercially available food items were utilized. In accordance with the individual energy requirements of each participant, the weight of each food item was determined with a precision of 0.1 g using a digital scale (OHAUS Explorer, OHAUS Europe). Additionally, the weight of any remaining food was also recorded. The individual diet composition, along with the actual energy and macronutrient intake, were calculated using the PRODI expert software (v.6.12, Wissenschaftliche Verlagsgesellschaft). The macronutrient content % was calculated using the following factors: 4 kcal g^−1^ protein or CHO, 9 kcal g^−1^ fat and 2 kcal g^−1^ fibre^7^. The energy density (kcal g^−1^) of the UPF diet was calculated using self-determined cooking loss factors for heated food items obtained during the study. Therefore, food items were weighed multiple times (2–10 times) before and following the heating process. The cooking loss factors were calculated by dividing the food weight before heating by the food weight following heating, and a mean value was determined for each food item. These cooking loss factors were then used to calculate the actual energy density of the food consumed. All food was provided, and participants were instructed to consume only the allocated foods, water and unsweetened fruit tea or peppermint tea. Additionally, they were asked to refrain from engaging in any vigorous exercise during the intervention periods.

### Energy expenditure and macronutrient oxidation

The two identical 9.8 m^2^ (24,282 l) WRICs at the Institute of Human Nutrition at Kiel University, each equipped with the Promethion model GA-3m2/FG-250 (Sable Systems International), were used in this study to assess total energy expenditure (TEE) and macronutrient oxidation. Further details regarding the equipment, methodology and technical and biological validation of the WRICs at Kiel University can be found elsewhere^49^. Regular quality checks were conducted throughout the study period (monthly to quarterly) using propane combustion tests and WRIC measurements were found to be within ±5% of the expected values. The rates of oxygen consumption (VO_2_) and carbon dioxide production (VCO_2_) were measured continuously at a flow rate of 80 l min^−1^, with mean values obtained from minute-to-minute intervals before metabolic calculations. Additionally, VO_2_ and VCO_2_ were corrected for urinary nitrogen excretion^50^ (see ‘Blood and urine sampling’ section). Energy expenditure was calculated using the Weir equation^51^ and macronutrient oxidation was calculated using the nonprotein respiratory quotient (npRQ) according to Jéquier and Felber^52^ from corrected VO_2_ and VCO_2_ from 7:15 to 7:15 the next day. The absolute energy and macronutrient balances (in kcal d^−1^) were determined by subtracting the macronutrient oxidation or EE from the respective intake. The relative energy balance (in %) was calculated as the percentage of EI relative to the respective TEE (TEE = EI/TEE × 100 – 100). To examine fuel utilization (energy partitioning), macronutrient oxidation as a percentage of TEE was calculated (macronutrient utilization (%) = 24-h oxidation (kcal d^−1^)/TEE (kcal d^−1^) × 100). To quantify whether there is a different metabolic basis of the positive energy balance between high-protein UPFs and normal-protein UPFs, a quotient of energy balance per nitrogen balance (kcal gN^−1^ d^−1^) was calculated as energy balance (kcal d^−1^) divided by nitrogen balance (g d^−1^). Sleeping energy expenditure (SEE) was calculated as reported by Schrauwen et al. from the lowest energy expenditure value of three consecutive hours during sleep between 24:00 and 7:14 (ref. ^53^). PAL was determined as TEE divided by REE (REE = SEE + SEE × 0.05).

### Blood and urine sampling

Blood samples could not be collected from one person, so results for blood parameters are shown for *n* = 20 individuals. Blood samples were collected after an overnight fast and 30, 60, 90, 120, 150 and 180 min postprandially after breakfast on day 5 per intervention (second day in the WRIC). Immediately after blood sampling, inhibitors were added to serum samples for measurement of acylated ghrelin and total PYY to prevent hormone degradation (Pefabloc SC, Roche Diagnostics and DPP-IV inhibitor, Merck). Plasma samples were centrifuged directly, and serum samples were centrifuged after clotting for 30 min at room temperature, both at 2,500*g* for 10 min. Concentrations of plasma glucose (hexokinase method, OSR6121, Beckman Coulter) and serum insulin (chemiluminescent immunoassay, Alinity Insulin Reagent kit 04T75, Abbott) were measured in all samples. Serum concentrations of acylated ghrelin (intra assay CV 5.5–10.3%; inter assay CV 5.9–10.9%; Biovendor) and total PYY (intra assay CV 6.1–8.5%; inter assay CV 5.5–10.3%, Yanaihara Institute) as well as plasma concentration of fibroblast growth factor 21 (FGF21; intra assay CV 1.6–2.4%; inter assay CV 3.1–3.5%; Biovendor) and glucagon (intra assay CV 2.1–14%; inter assay CV 7.0–16%, Mercodia) were quantified at 0, 30, 60, 120 and 180 min using commercial ELISA assays. The sample analyses were performed at the ‘German Institute of Human Nutrition’, Potsdam-Rehbrücke, Department of Nutrition and Gerontology, and an accredited and certified laboratory (Labor Dr. Krause und Kollegen) in Kiel, Germany. Total area under the curve (tAUC) or incremental area under the curve (iAUC) for blood parameters were calculated for 3 h using the trapezoidal rule. The insulin:glucagon molar ratio was calculated as insulin (µU ml^−1^)/glucagon (pg ml^−1^) × 23.3 according to Seitz et al.^29^. Interstitial glucose levels were continuously monitored throughout the study period (FreeStyle Libre 2, Abbott Diabetes Care) and diurnal glycaemia was calculated as tAUC for 23.75 h. In addition, basal glycaemia was calculated as tAUC from 24:00 to 7:00.

On days 4 and 5 (days in WRIC), participants collected 24-h urine from the second void until the first void the next morning, which was stored in a refrigerator until final sampling after 24 h. Urea excretion was assessed photometrically in 24-h urine to calculate nitrogen (N) excretion (1 g urea contains 46.7% N). Urinary non-urea-N excretion was estimated as +0.031 g N × body weight (kg) and obligate N losses by faeces and skin were assumed to be +2.5 g N d^−1^. Total N excretion (N_excr_) was thus calculated as N_excr_ (g) = 0.467 × urea excretion (g) + 0.031 × body weight (kg) + 2.5 (g). Nitrogen balance (g d^−1^) was calculated as nitrogen intake from dietary protein minus urinary nitrogen excretion. To assess diurnal insulin secretion, 24-h urinary C-peptide excretion was measured by electrochemiluminescence immunoassay. Aliquots of all samples were stored at −40 °C until analysis.

### Subjective appetite

On day 5 per intervention, appetite ratings were assessed with pen and paper using a 100-mm visual analogue scale (VAS) for sensations of hunger, fullness, desire to eat and prospective food consumption at 0, 30, 60, 90, 120, 150 and 180 min postprandially after breakfast. The VAS consisted of a 100-mm horizontal line anchored with ‘not at all’ at 0 mm and ‘extremely’ at 100 mm. A composite appetite score for each sensation was calculated as the average of all appetite ratings (fullness reversed) per time point according to Beaulieu et al.^54^. The iAUC was calculated for 3 h for all sensations and the composite appetite score.

### Eating rate

All meals on days 4 and 5 were webcam recorded to assess individual meal duration and number of bites and chews per meal. Participants were unaware of the parameters of interest, but were told that the purpose of the video recording was to monitor that they did not use the phone or other media during mealtimes to avoid distracted eating. Eating rate (g min^−1^) and EI rate (kcal min^−1^) were calculated by dividing the weight of food consumed per meal or the EI per meal by the duration of each meal. Mean eating rate (g min^−1^) and EI rate (kcal min^−1^) per meal, as well as mean number of bites per meal and chewing frequency (chews per bite) were calculated from six individual meals per person.

### Gastric emptying

To determine gastric emptying, a ^13^C-gastric emptying breath test (^13^C-GEBT) was conducted on day 6 following a standardized isocaloric test meal (porridge, 25% of individual REE) labelled with 100 mg ^13^C-sodium acetate (Hanseaten Apotheke). The test meal had to be ingested within 5–10 min and breath samples were collected in gas-tight, aluminized breath bags at baseline and 15, 30, 45, 60, 75, 90, 105, 120, 150, 180, 210 and 240 min postprandially. During sampling, participants remained sedentary and water consumption was limited to 1 l. To minimize potential intraindividual dilution effects, participants maintained a drinking protocol during the first test and repeated it during the second test. Breath samples were analysed in duplicate using a non-dispersive, isotope-selective infra-red isotope analyser (IRIS DOC 2, Kibion) no later than 1 week after breath sampling. Percentage ^13^C-dose recovery (PDR in % per hour) was calculated from delta values (o/oo) from which gastric half-emptying time (*t*_1/2_), gastric emptying lag time (*t*_lag_) and gastric emptying coefficient were calculated according to the method of Ghoos^55^ and as previously described by our group^56^.

### Body composition

Before the study began, body weight was measured in underwear to the nearest 0.1 kg using an electronic scale (seca 285, seca) and fat mass was determined by quantitative magnetic resonance (ECOMRI-AH, Echo Medical Systems). Fat mass index was calculated as fat mass divided by height squared (kg m^−2^).

### Statistical analysis

A required sample size of *n* = 22 (independent of sex) was calculated using G*Power v.3.1.9.7 software (written by F. Faul, Kiel University) to detect a difference of 25% (2.41 MJ d^−1^) in ad libitum EI between a normal versus high-protein diet (15% versus 30% energy from protein) according to Martens et al.^57^ by applying a two-sided matched pairs *t*-test (assuming a power of 95 % and an α-level of 5%). The primary outcome was energy balance (ad libitum EI and energy expenditure) at intervention days in the WRIC. Secondary outcomes were eating rate (kcal min^−1^ and g min^−1^) and chewing frequency (chews per bite), levels of the appetite-related hormones ghrelin and PYY, subjective appetite perceptions (assessed by VAS), gastric emptying, macronutrient oxidation and glycaemia (for 24 h and postprandially). The limited sample size precludes subgroup analyses with regard to sex or ethnicity.

Data were analysed using the SPSS software package (SPSS Statistics for Windows, IBM v.29.0). The assumption of normality was verified using the Kolmogorov–Smirnov test. Differences between high-protein and normal-protein interventions were analysed by two-sided paired *t*-test for normally distributed variables or by Wilcoxon test for nonparametric variables, as appropriate. Relationships between normally distributed variables were determined using Pearson correlation coefficients. Graphs were plotted using GraphPad Prism v.10.2.2 (GraphPad Prism for Windows, GraphPad Software). Data are presented as mean ± s.d. and a two-sided *P* < 0.05 was considered to be statistically significant.

Three individuals were excluded from the study due to premature termination, and their data are only included in the baseline characteristics (Extended Data Table 1). No outliers were excluded from the data analysis and thus, no sensitivity analysis was performed. In cases of missing data, the respective participant was excluded from the corresponding analysis. The numbers of participants included in the evaluations are indicated in the figure legends.

### Reporting summary

Further information on research design is available in the Nature Portfolio Reporting Summary linked to this article.

## Supplementary information

Supplementary Information Supplementary Figs. 1 and 2 and Table 1.

Reporting Summary

## Source data

Source Data Fig. 1 Source data.

Source Data Fig. 2 Source data.

Source Data Fig. 3 Source data.

Source Data Extended Data Fig. 3 Source data.

Source Data Extended Data Fig. 4 Source data.

## Figures and Tables

**Extended Data Table 1:**
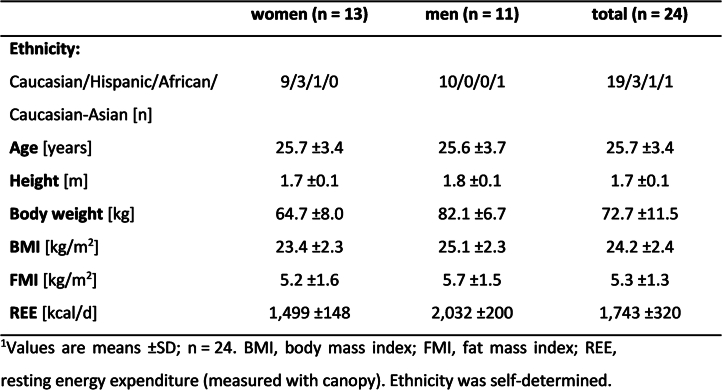
Baseline anthropometrics of the study population^1^

## Data Availability

The data that support the plots within this paper have been provided as Source Data. Other data of this study are available from the corresponding author upon reasonable request. The data that support the findings of this study are not publicly available due to the data privacy statement in the participant information form. Source data are provided with this paper.
