# Supplementary material for: Short-term effects of high-protein, lower-carbohydrate ultra-processed foods on human energy balance
Source: Nat Metab. 2025 Mar 13;7(4):704–13. doi: 10.1038/s42255-025-01247-4 (PMC12021659; doi:10.1038/s42255-025-01247-4)
Supplement: Supplementary file 1 — Supplementary Figs. 1 and 2 and Table 1. [file 42255_2025_1247_MOESM1_ESM.pdf]

nature metabolism

Supplementary information

https://doi.org/10.1038/s42255-025-01247-4

Short-term effects of high-protein, lower-carbohydrate ultra-processed foods on human energy balance

In the format provided by the authors and unedited

Content

Supplementary Figure 1: Meals provided to the subjects on days 4 and 5 4

Supplementary Figure 2: Exemplary meals provided to the subjects during run-in period 5

Supplementary Table 1: CONSORT checklist of information to include when reporting randomised crossover trials 6

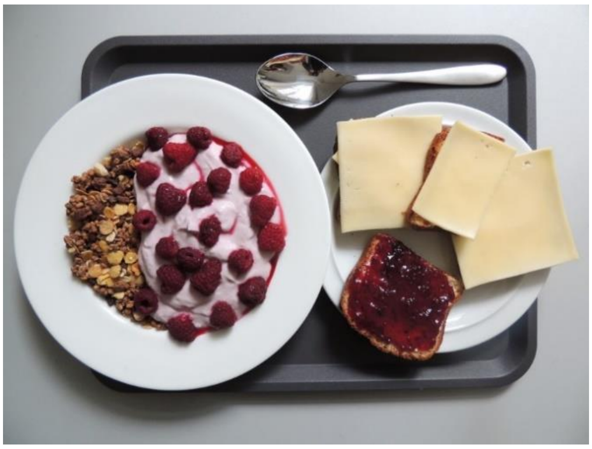

Breakfast day 4 HPLC
high protein cereals (Dr. Oetker), *high protein* raspberry-pomegranate yoghurt (Ehrmann) with raspberries, *protein* toast (BenFit), butter, cheese, jam

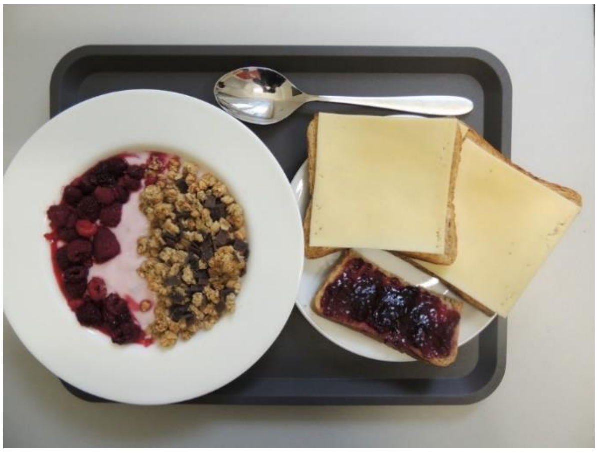

Breakfast day 4 NPNC
cereals (Dr. Oetker), raspberry yoghurt (Ehrmann), with raspberries, toast (Harry Brot), butter, cheese, jam

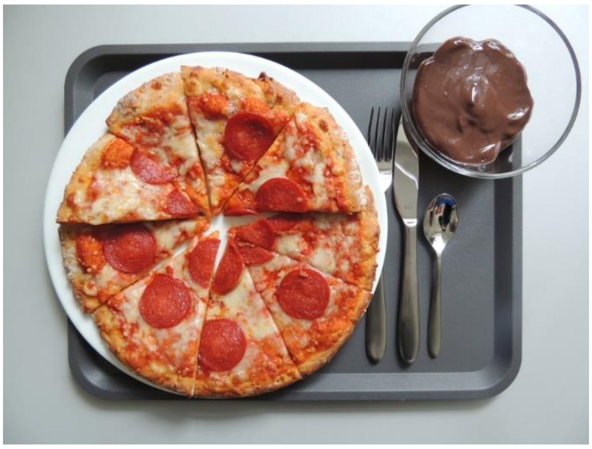

Lunch day 4 HPLC
high protein Salami pizza (Prozis), *high protein* chocolate pudding (Ehrmann)

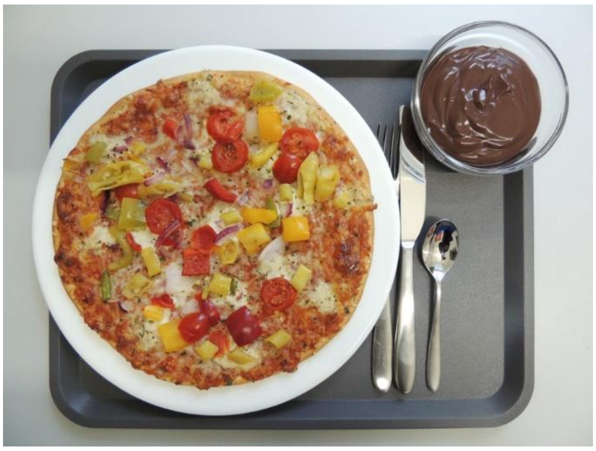

Lunch day 4 NPNC
vegetable pizza (Dr. Oetker), plant-based chocolate pudding (Dr. Oetker)

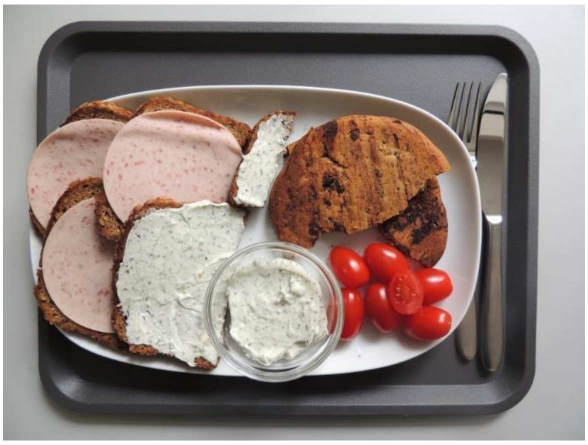

Dinner day 4 HPLC
protein bread (Harry Brot), cream cheese (Mondelez), ham sausage (Rügenwalder Mühle), tomatoes, *protein* chocolate chip cookie (Foodspring)

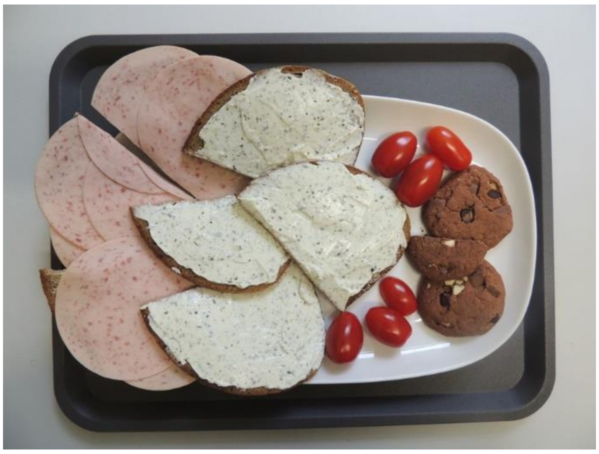

Dinner day 4 NPNC
bread (Harry Brot), cream cheese (Mondelez), ham sausage (Rügenwalder Mühle), tomatoes, chocolate chip cookie (Rewe Beste Wahl)

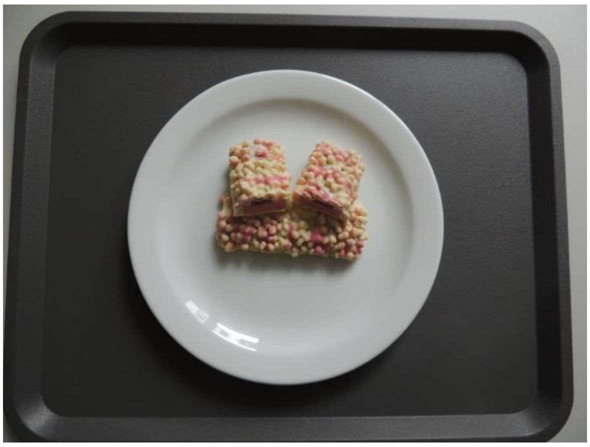

Snack day 4 HPLC
protein bar raspberry crisp (dm Sportness)

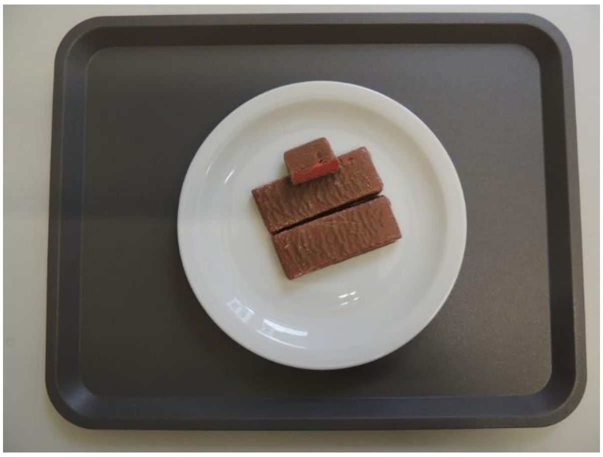

Snack day 4 NPNC
energy bar raspberry yoghurt (dm Sportness)

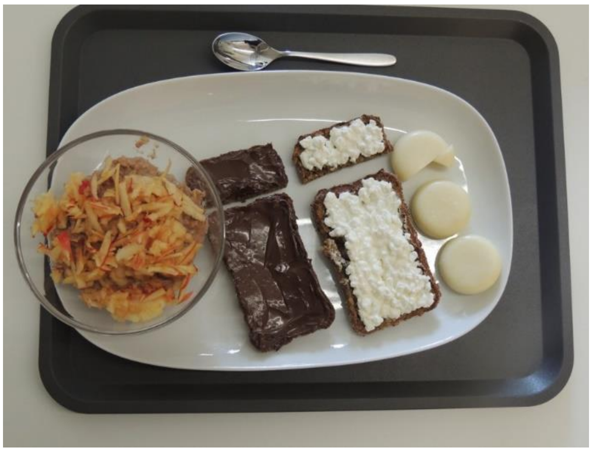

Breakfast day 5 HPLC
protein chocolate porridge (Seitenbacher) with milk (3.5 % fat) and apple, *protein* toast buns (Mestemacher), cream cheese with herbs (Mondelez), *high protein* cheese (Mini Babybel), *protein* hazelnut spread (Foodspring)

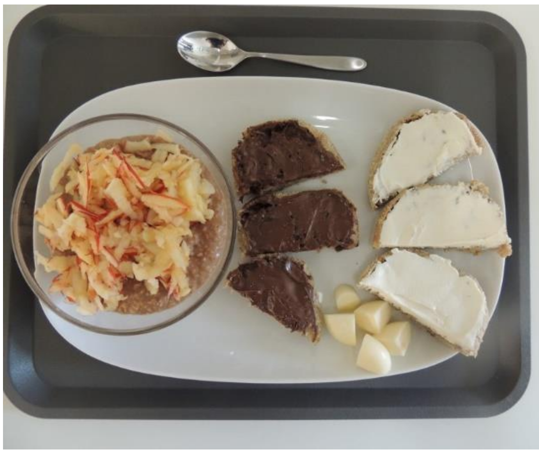

Breakfast day 5 NPNC
chocolate porridge (Kölln) with milk (1.5 % fat), water and apple, seeded toast buns (Harry Brot), cream cheese (Mondelez), cheese (Mini Babybel), hazelnut spread (Ferrero)

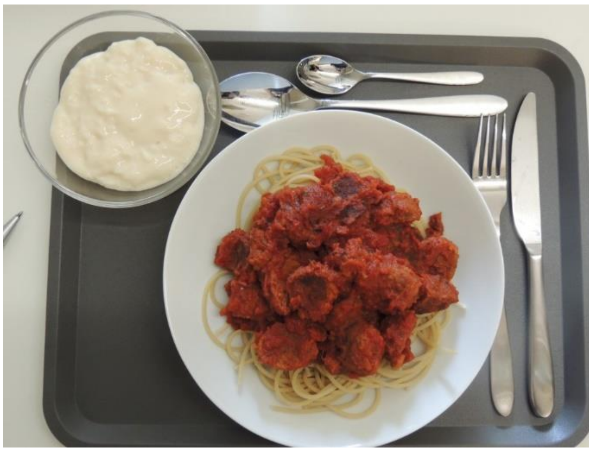

Lunch day 5 HPLC
protein spaghetti (Prozis) with with tomato sauce (Barilla) and vegetarian dumplings (Garden Gourmet, Nestlé), *high protein* rice pudding (Müller Milch) and red fruit jelly (Zum Dorfkrug)

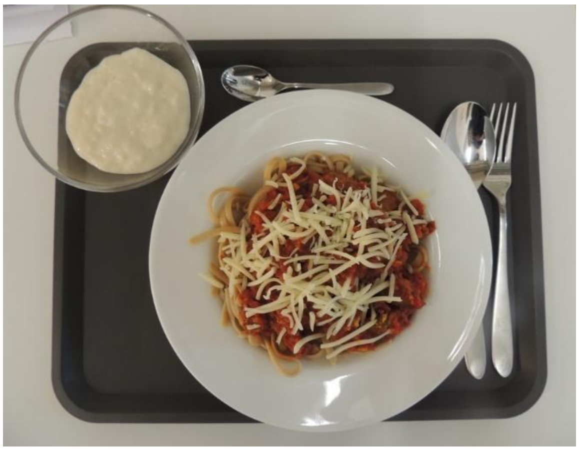

Lunch day 5 NPNC
whole grain spaghetti (Barilla) with tomato sauce (Barilla), cheese (Milram) and vegan falafel (Garden Gourmet, Nestlé), rice pudding (Müller Milch) and red fruit jelly (Zum Dorfkrug)

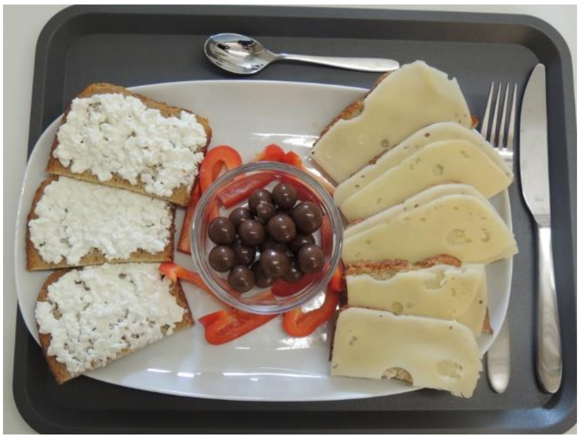

Dinner day 5 HPLC
high protein bread (Mestemacher), butter, cheese (Grünländer), cream cheese (Mondelez), bell pepper, *high protein* crispy balls (Ehrmann)

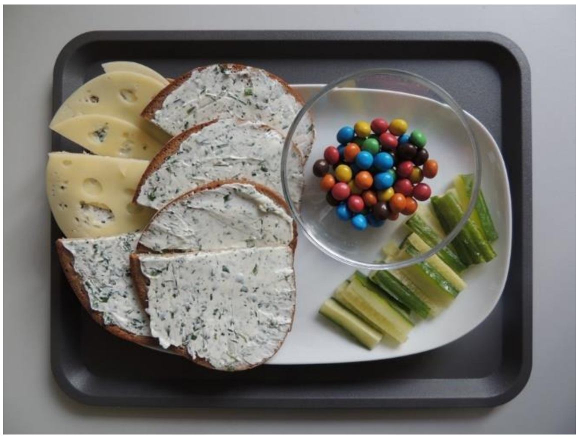

Dinner day 5 NPNC
bread (Harry Brot), cheese (Grünländer), cream cheese (Mondelez), herbs, cucumber, M&M's crispy (Mars)

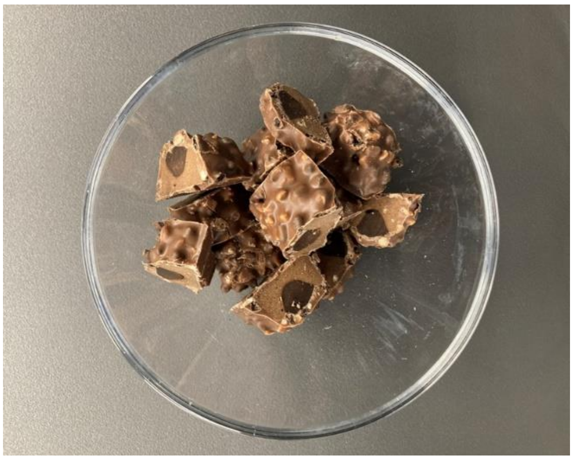

snack day 5 HPLC
protein bar hazelnut nougat crisp (dm Sportness)

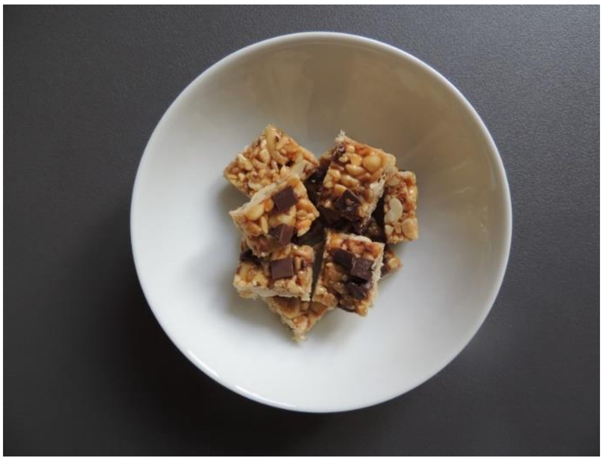

snack day 5 NPNC
nut bar (Corny)

Supplementary Figure 1: Meals provided to the subjects on days 4 and 5

During intervention days within the whole-room indirect calorimeter (WRIC), an ultra-processed diet (>80 % ultra-processed food) was presented to the subjects. A high protein-lower carbohydrate diet (HPLC, 30 % protein, 29 % carbohydrates, CHO) and a normal protein-normal CHO diet (NPNC, 13 % protein, 46 % CHO) were received in randomized order. Subjects were blinded to the protein content of the diet. Breakfast (08:30), lunch (13:30), dinner (18:30) and an optional evening snack (20:00-21:15) were provided. The photographs of the daily menus described above show exemplary meals. The actual amount of food presented to the subjects depended on individual energy requirements as described in the methods section in the chapter "diet composition" of the main text.

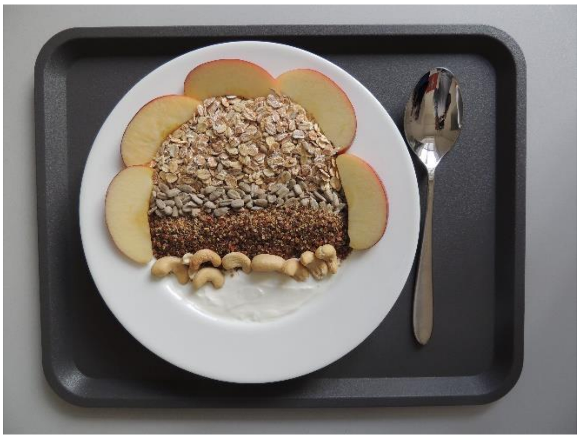

Breakfast HPLC
whole grain cereals with sunflower seeds, cashews and flaxseed, skyr (Arla) and apple

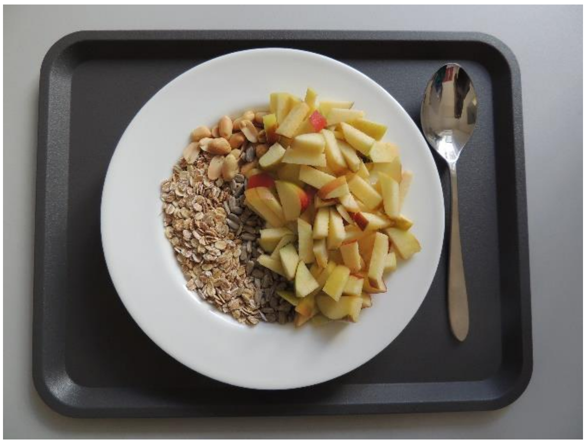

Breakfast NPNC
whole grain cereals with sunflower seeds, yoghurt and Greek yoghurt, apple

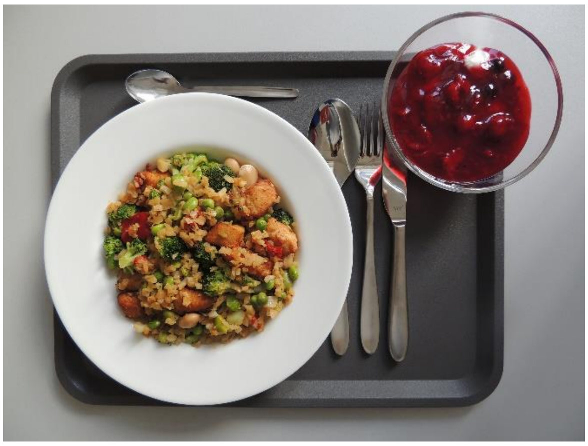

Lunch HPLC
high protein vegetable lentil curry (Frosta) with edamame (peeled) and red fruit jelly (Zum Dorfkrug)

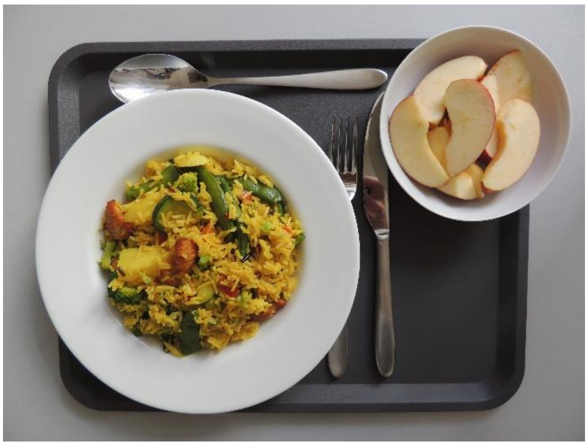

Lunch NPNC
chicken curry with rice and vegetables (Frosta), broccoli, apple

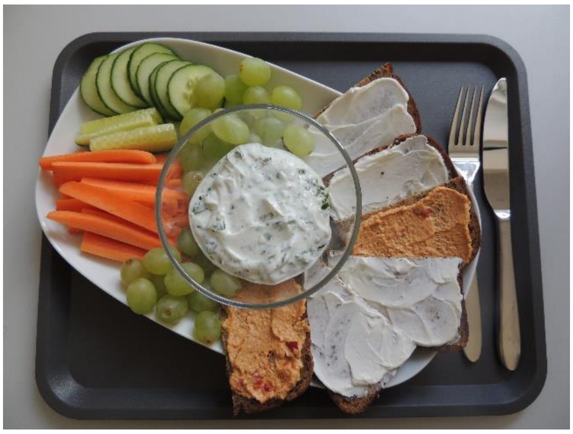

Dinner HPLC
protein bread (dm), cream cheese (Mondelez), hummus (Noa), grapes, cucmber, carrott, curd and Greek yoghurt with herbs

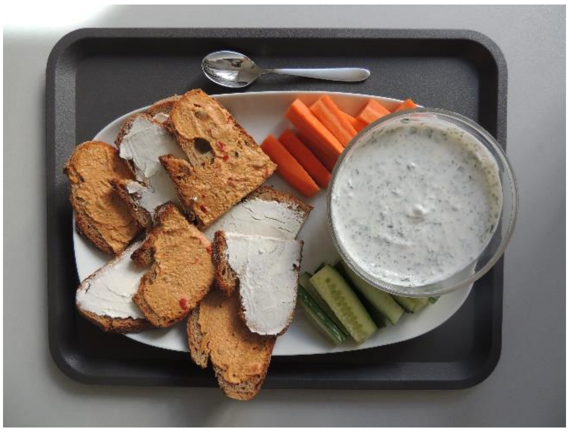

Dinner NPNC
bread (Harry Brot), cream cheese (Mondelez), Mediterranean vegetable spread, cucmber, carrott, curd and Greek yoghurt with herbs

Supplementary Figure 2: Exemplary meals provided to the subjects during run-in period

During the run-in period with controlled nutrition at home, a diet with <45 % ultra-processed food was presented to the subjects. A high protein-lower carbohydrate diet (HPLC, 30 % protein, 29 %

carbohydrates, CHO) and a normal protein-normal CHO diet (NPNC, 13 % protein, 46 % CHO) were received in randomized order. Subjects were blinded to the protein content of the diet. Breakfast, lunch and dinner were provided to eat at home, no other foods or caloric drinks were allowed. The photographs of the daily menus described above show exemplary meals. The actual amount of food presented to the subjects depended on individual energy requirements as described in the methods section in the chapter "diet composition" of the main text.

Supplementary Table 1: CONSORT checklist of information to include when reporting randomised crossover trials

**Section/topic**	**Item No**	**Description**	**Page No***
Title†	1a	Identification as a randomised crossover trial in the title	-
Abstract†	1b	Specify a crossover design and report all information outlined in Table 2 of Dwan et al, *BMJ* 2019;366:l4378; doi: 10.1136/bmj.l4378	2
Introduction:			
Background‡	2a	Scientific background and explanation of rationale	3-4
Objectives‡	2b	Specific objectives or hypotheses	3-4
**Methods:**			
Trial design†	3a	Rationale for a crossover design. Description of the design features including allocation ratio, especially the number and duration of periods, duration of washout period, and consideration of carry over effect	18-19
Change from protocol‡	3b	Important changes to methods after trial commencement (such as eligibility criteria), with reasons	na
Participants‡	4a	Eligibility criteria for participants	17-18
Settings and location‡	4b	Settings and locations where the data were collected	17
Interventions†	5	The interventions with sufficient details to allow replication, including how and when they were actually administered	18-22
Outcomes‡	6a	Completely defined prespecified primary and secondary outcome measures, including how and when they were assessed	27, 20-27
Changes to outcomes‡	6b	Any changes to trial outcomes after the trial commenced, with reasons	na
Sample size†	7a	How sample size was determined, accounting for within participant variability	27
Interim analyses and stopping guidelines‡	7b	When applicable, explanation of any interim analyses and stopping guidelines	na
**Randomisation:**			
Sequence generation‡	8a	Method used to generate the random allocation sequence	18
Sequence generation‡	8b	Type of randomisation; details of any restriction (such as blocking and block size)	18
Allocation concealment‡	9	Mechanism used to implement the random allocation sequence§ (such as sequentially numbered mechanism containers),	18-19

		describing any steps taken to conceal the sequence until interventions were assigned	
Implementation†	10	Who generated the random allocation sequence,§ who enrolled participants, and who assigned participants to the sequence of interventions	18
Blinding‡	11a	If done, who was blinded after assignment to interventions (for example, participants, care providers, those assessing outcomes) and how	19
Similarity of interventions‡	11b	If relevant, description of the similarity of interventions	na
Statistical methods†	12a	Statistical methods used to compare groups for primary and secondary outcomes which are appropriate for crossover design (that is, based on within participant comparison)	27
Additional analyses‡	12b	Methods for additional analyses, such as subgroup analyses and adjusted analyses	na
**Results**			
Participant flow (a diagram is recommended)†	13a	The numbers of participants who were randomly assigned, received intended treatment, and were analysed strongly for the primary outcome, separately for each sequence and period	Ext. Data Fig. 1
Losses and exclusions†	13b	No of participants excluded at each stage, with reasons, separately for each sequence and period	Ext. Data Fig. 1
Recruitment‡	14a	Dates defining the periods of recruitment and follow-up	17
Trial end‡	14b	Why the trial ended or was stopped	na
Baseline data†	15	A table showing baseline demographic and clinical characteristics by sequence and period	Ext. Data Table 1
Numbers analysed†	16	Number of participants (denominator) included in each analysis and whether the analysis was by original assigned groups	Ext. Data Fig. 1
Outcomes and estimation†	17a	For each primary and secondary outcome, results including estimated effect size and its precision (such as 95% confidence interval) should be based on within participant comparisons.¶ In addition, results for each intervention in each period are recommended	4-11
Binary outcomes‡	17b	For binary outcomes, presentation of both absolute and relative effect sizes is recommended	na
Ancillary analyses‡	18	Results of any other analyses performed, including subgroup analyses and adjusted analyses, distinguishing prespecified from exploratory	na
Harms†	19	Describe all important harms or untended effects in a way that accounts for the design (for specific guidance, see CONSORT for harms[¥])	na
**Discussion:**			
Limitations†	20	Trial limitations, addressing sources of potential bias, imprecision, and if relevant, multiplicity of analyses. Consider potential carry over effects	10-11
Generalisability‡	21	Generalisability (external validity, applicability) of the trial findings	11
Interpretation‡	22	Interpretation consistent with results, balancing benefits and harms, and considering other relevant evidence	10-11
**Other information:**			

Registration‡	23	Registration number and name of trial registry	17
Protocol‡	24	Where the full trial protocol can be accessed, if available	na
Funding‡	25	Sources of funding and other support (such as supply of drugs), role of funders	12

CONSORT=Consolidated Standards of Reporting Trials.
*Note: page numbers are optional depending on journal requirements.
†Modified original CONSORT item.
‡Unmodified CONSORT item.
§Random sequence here refers to a list of random orders, typically generated through a computer program. This should not be confused with the sequence of interventions in a randomised crossover trial, for example receiving intervention A before B for an individual trial participant.
¶A within participant comparison takes into account the correlation between measurements for each participant because they act as their own control, therefore measurements are not independent.
¥ Ioannidis JP, Evans SJ, Gotzsche PC, et al. CONSORT Group.Better reporting of harms in randomized trials: an extension of the CONSORT statement. *Ann Intern Med* 2004;141:781-8. doi:10.7326/0003-4819-141-10-200411160-00009
