# Supplementary material for: Short-term effects of high-protein, lower-carbohydrate ultra-processed foods on human energy balance
Source: Nat Metab. 2025 Mar 13;7(4):704–13. doi: 10.1038/s42255-025-01247-4 (PMC12021659; doi:10.1038/s42255-025-01247-4)
Supplement: Supplementary file 2 — Reporting Summary [file 42255_2025_1247_MOESM2_ESM.pdf]

nature portfolio

Corresponding author(s): Anja Bosy-Westphal

Last updated by author(s): Jan 28, 2025

Reporting Summary

Nature Portfolio wishes to improve the reproducibility of the work that we publish. This form provides structure for consistency and transparency in reporting. For further information on Nature Portfolio policies, see our Editorial Policies and the Editorial Policy Checklist.

Statistics

For all statistical analyses, confirm that the following items are present in the figure legend, table legend, main text, or Methods section.

n/a	Confirmed	
☐	☒	The exact sample size (n) for each experimental group/condition, given as a discrete number and unit of measurement
☐	☒	A statement on whether measurements were taken from distinct samples or whether the same sample was measured repeatedly
☐	☒	The statistical test(s) used AND whether they are one- or two-sided<br>*Only common tests should be described solely by name; describe more complex techniques in the Methods section.*
☒	☐	A description of all covariates tested
☐	☒	A description of any assumptions or corrections, such as tests of normality and adjustment for multiple comparisons
☐	☒	A full description of the statistical parameters including central tendency (e.g. means) or other basic estimates (e.g. regression coefficient) AND variation (e.g. standard deviation) or associated estimates of uncertainty (e.g. confidence intervals)
☐	☒	For null hypothesis testing, the test statistic (e.g. F, t, r) with confidence intervals, effect sizes, degrees of freedom and P value noted<br>*Give P values as exact values whenever suitable.*
☒	☐	For Bayesian analysis, information on the choice of priors and Markov chain Monte Carlo settings
☒	☐	For hierarchical and complex designs, identification of the appropriate level for tests and full reporting of outcomes
☐	☒	Estimates of effect sizes (e.g. Cohen's d, Pearson's r), indicating how they were calculated

Our web collection on statistics for biologists contains articles on many of the points above.

Software and code

Policy information about availability of computer code

Data collection	CaloScreen version 1.3.16, Sable Systems International, Las Vegas, USA.
Data analysis	Microsoft Excel 2016. PRODI expert software, version 6.12, Wissenschaftliche Verlagsgesellschaft Stuttgart, Germany. ExpeData version 1.9.27, Sable Systems International, Las Vegas, USA. G*Power 3.1.9.7 software (written by F. Faul, University of Kiel in Germany). SPSS software package, version 29.0, IBM Corp. IBM SPSS Statistics for Windows, Armonk, NY: IBM Corp. GraphPad Prism 10.2.2, GraphPad Prism for Windows, GraphPad Software, La Jolla, California USA.

For manuscripts utilizing custom algorithms or software that are central to the research but not yet described in published literature, software must be made available to editors and reviewers. We strongly encourage code deposition in a community repository (e.g. GitHub). See the Nature Portfolio guidelines for submitting code & software for further information.

Data

Policy information about availability of data

All manuscripts must include a data availability statement. This statement should provide the following information, where applicable:

- Accession codes, unique identifiers, or web links for publicly available datasets
- A description of any restrictions on data availability
- For clinical datasets or third party data, please ensure that the statement adheres to our policy

The data that support the findings of this study are not publicly available due to the data privacy statement in the subject information form. The data that support the plots within this paper have been provided as Source Data. Other data of this study are available from the corresponding author upon reasonable request.

Research involving human participants, their data, or biological material

Policy information about studies with human participants or human data. See also policy information about sex, gender (identity/presentation), and sexual orientation and race, ethnicity and racism.

Reporting on sex and gender	Sex of the study participants was determined by self-report. The objective was to achieve an equal distribution of sexes based on self-reports. As the study was not designed to detect sex differences, these were not analyzed.
Reporting on race, ethnicity, or other socially relevant groupings	The majority of participants had Caucasian origins, but three persons had Hispanic, one person had African and one person had Caucasian-Asian origins. All information is provided in the Extended Data Table 1.
Population characteristics	Twenty-four healthy young adults (13 women, 11 men) were investigated. Basal characteristics of the study population can be found in the Extended Data Table 1.
Recruitment	Healthy young adults between the ages of 18 and 35 years with a BMI between 19 and 29 kg/m2 and a low-to-normal level of habitual physical activity who voluntarily signed up were recruited and data were collected from May 2022 to April 2023 at the Kiel University in Germany using notice board, and social media postings, information on the website of Kiel University and by contacting former study participants who have consented to this. Women were included with a regular menstrual cycle only and during their follicular phase to avoid influences of the female cycle on EE and appetite.<br>Exclusion criteria were: chronic diseases (including renal dysfunction), regular use of medication, alternative eating habits (vegan, vegetarian, etc.), food allergies or intolerances, claustrophobia, smoking, high habitual physical activity (≥1 h/d), current weight loss diet or weight loss of >5 kg in the last 3 months, pregnancy or lactation.<br>Prior to their enrollment in the study, participants were presented with the proposed meal plans. Only those who expressed a willingness to adhere to the specified dietary regimen were included in the study.
Ethics oversight	The study protocol was approved by the ethics committee of the Medical Faculty at Kiel University, Germany (D456/22) in accordance with the Declaration of Helsinki. This trial was registered at clinicaltrials.gov as NCT05337007. All subjects provided written informed consent before participation.

Note that full information on the approval of the study protocol must also be provided in the manuscript.

Field-specific reporting

Please select the one below that is the best fit for your research. If you are not sure, read the appropriate sections before making your selection.

☒ Life sciences ☐ Behavioural & social sciences ☐ Ecological, evolutionary & environmental sciences

For a reference copy of the document with all sections, see nature.com/documents/nr-reporting-summary-flat.pdf

Life sciences study design

All studies must disclose on these points even when the disclosure is negative.

Sample size	A required sample size of n = 22 (independent of sex) was calculated using G*Power 3.1.9.7 software (written by F. Faul, University of Kiel in Germany) to detect a difference of 25 % (2.41 MJ/d) in ad libitum energy intake between a normal vs. high-protein diet (15 % vs. 30 % energy from protein) according to Martens et al.65 by applying a two-sided matched pairs t-test (assuming a power of 95 % and an α-level of 5 %).
Data exclusions	Three subjects were excluded from the analysis due to premature discontinuation for personal reasons including spontaneous scheduling difficulties, resulting in a final population of 21 subjects.
Replication	Regular quality checks of whole room indirect calorimeter (WRIC) measurements were conducted throughout the study period (monthly to quarterly) using propane combustion tests. Measurements of VO2, VCO2 and RQ (VCO2/VO2) were found to be within ±5 % of the expected values for all tests.
Randomization	The order of NPNC and HPLC intervention was randomized in a chronological sequence. The sequence of interventions was determined through block randomization, with computer-generated random numbers and a block size of 12. The randomization was stratified by sex and

	the allocation to WRIC 1 or 2.
Blinding	The participants were not made aware of the specific sequence in which they would receive the interventions (single-blind design).

Behavioural & social sciences study design

All studies must disclose on these points even when the disclosure is negative.

Study description	*Briefly describe the study type including whether data are quantitative, qualitative, or mixed-methods (e.g. qualitative cross-sectional, quantitative experimental, mixed-methods case study).*
Research sample	*State the research sample (e.g. Harvard university undergraduates, villagers in rural India) and provide relevant demographic information (e.g. age, sex) and indicate whether the sample is representative. Provide a rationale for the study sample chosen. For studies involving existing datasets, please describe the dataset and source.*
Sampling strategy	*Describe the sampling procedure (e.g. random, snowball, stratified, convenience). Describe the statistical methods that were used to predetermine sample size OR if no sample-size calculation was performed, describe how sample sizes were chosen and provide a rationale for why these sample sizes are sufficient. For qualitative data, please indicate whether data saturation was considered, and what criteria were used to decide that no further sampling was needed.*
Data collection	*Provide details about the data collection procedure, including the instruments or devices used to record the data (e.g. pen and paper, computer, eye tracker, video or audio equipment) whether anyone was present besides the participant(s) and the researcher, and whether the researcher was blind to experimental condition and/or the study hypothesis during data collection.*
Timing	*Indicate the start and stop dates of data collection. If there is a gap between collection periods, state the dates for each sample cohort.*
Data exclusions	*If no data were excluded from the analyses, state so OR if data were excluded, provide the exact number of exclusions and the rationale behind them, indicating whether exclusion criteria were pre-established.*
Non-participation	*State how many participants dropped out/declined participation and the reason(s) given OR provide response rate OR state that no participants dropped out/declined participation.*
Randomization	*If participants were not allocated into experimental groups, state so OR describe how participants were allocated to groups, and if allocation was not random, describe how covariates were controlled.*

Ecological, evolutionary & environmental sciences study design

All studies must disclose on these points even when the disclosure is negative.

Study description	*Briefly describe the study. For quantitative data include treatment factors and interactions, design structure (e.g. factorial, nested, hierarchical), nature and number of experimental units and replicates.*
Research sample	*Describe the research sample (e.g. a group of tagged Passer domesticus, all Stenocereus thurberi within Organ Pipe Cactus National Monument), and provide a rationale for the sample choice. When relevant, describe the organism taxa, source, sex, age range and any manipulations. State what population the sample is meant to represent when applicable. For studies involving existing datasets, describe the data and its source.*
Sampling strategy	*Note the sampling procedure. Describe the statistical methods that were used to predetermine sample size OR if no sample-size calculation was performed, describe how sample sizes were chosen and provide a rationale for why these sample sizes are sufficient.*
Data collection	*Describe the data collection procedure, including who recorded the data and how.*
Timing and spatial scale	*Indicate the start and stop dates of data collection, noting the frequency and periodicity of sampling and providing a rationale for these choices. If there is a gap between collection periods, state the dates for each sample cohort. Specify the spatial scale from which the data are taken*
Data exclusions	*If no data were excluded from the analyses, state so OR if data were excluded, describe the exclusions and the rationale behind them, indicating whether exclusion criteria were pre-established.*
Reproducibility	*Describe the measures taken to verify the reproducibility of experimental findings. For each experiment, note whether any attempts to repeat the experiment failed OR state that all attempts to repeat the experiment were successful.*
Randomization	*Describe how samples/organisms/participants were allocated into groups. If allocation was not random, describe how covariates were controlled. If this is not relevant to your study, explain why.*
Blinding	*Describe the extent of blinding used during data acquisition and analysis. If blinding was not possible, describe why OR explain why blinding was not relevant to your study.*

Did the study involve field work? ☐ Yes ☒ No

Reporting for specific materials, systems and methods

We require information from authors about some types of materials, experimental systems and methods used in many studies. Here, indicate whether each material, system or method listed is relevant to your study. If you are not sure if a list item applies to your research, read the appropriate section before selecting a response.

Materials & experimental systems

n/a	Involved in the study
☒	☐ Antibodies
☒	☐ Eukaryotic cell lines
☒	☐ Palaeontology and archaeology
☒	☐ Animals and other organisms
☐	☒ Clinical data
☒	☐ Dual use research of concern
☒	☐ Plants

Methods

n/a	Involved in the study
☒	☐ ChIP-seq
☒	☐ Flow cytometry
☒	☐ MRI-based neuroimaging

Clinical data

Policy information about clinical studies

All manuscripts should comply with the ICMJE guidelines for publication of clinical research and a completed CONSORT checklist must be included with all submissions.

Clinical trial registration: This trial was registered at clinicaltrials.gov as NCT05337007.

Study protocol: The study protocol is provided with the article.

Data collection: Data collection took place from May 2022 to April 2023 at the Institute of Human Nutrition, University of Kiel, Germany.

Outcomes: Primary and secondary outcome measures have been pre-definied in the clinical trial registration. The primary outcome was energy balance (ad libitum energy intake and energy expenditure) at intervention days in the WRIC. Ad libitum energy intake was assessed by weighing food before and after the meals. The individual diet composition, along with the actual energy and macronutrient intake, were calculated using the PRODI expert software (version 6.12, Wissenschaftliche Verlagsgesellschaft Stuttgart, Germany). Energy expenditure was calculated using the Weir equation from VO2 and VCO2 measured in a whole-room indirect calorimeter. The absolute energy balance (in kcal/d) was determined by subtracting the energy expenditure from energy intake. The relative energy balance (EB in %) was calculated as the percentage of energy intake (EI) relative to the respective TEE (TEE = EI / TEE x 100 – 100).

Plants

Seed stocks: *Report on the source of all seed stocks or other plant material used. If applicable, state the seed stock centre and catalogue number. If plant specimens were collected from the field, describe the collection location, date and sampling procedures.*

Novel plant genotypes: *Describe the methods by which all novel plant genotypes were produced. This includes those generated by transgenic approaches, gene editing, chemical/radiation-based mutagenesis and hybridization. For transgenic lines, describe the transformation method, the number of independent lines analyzed and the generation upon which experiments were performed. For gene-edited lines, describe the editor used, the endogenous sequence targeted for editing, the targeting guide RNA sequence (if applicable) and how the editor was applied.*

Authentication: *Describe any authentication procedures for each seed stock used or novel genotype generated. Describe any experiments used to assess the effect of a mutation and, where applicable, how potential secondary effects (e.g. second site T-DNA insertions, mosiacism, off-target gene editing) were examined.*
